# A High-Fat Diet Disrupts Nerve Lipids and Mitochondrial Function in Murine Models of Neuropathy

**DOI:** 10.3389/fphys.2022.921942

**Published:** 2022-08-22

**Authors:** Amy E. Rumora, Kai Guo, Lucy M. Hinder, Phillipe D. O’Brien, John M. Hayes, Junguk Hur, Eva L. Feldman

**Affiliations:** ^1^ Department of Neurology, University of Michigan, Ann Arbor, MI, United States; ^2^ Department of Neurology, Columbia University, New York, NY, United States; ^3^ Department of Biomedical Sciences, University of North Dakota, Grand Forks, ND, United States

**Keywords:** dyslipidemia, prediabetes, mitochondria, obesity, neuropathy, lipidomics, high-fat diet, metabolic syndrome

## Abstract

As the prevalence of prediabetes and type 2 diabetes (T2D) continues to increase worldwide, accompanying complications are also on the rise. The most prevalent complication, peripheral neuropathy (PN), is a complex process which remains incompletely understood. Dyslipidemia is an emerging risk factor for PN in both prediabetes and T2D, suggesting that excess lipids damage peripheral nerves; however, the precise lipid changes that contribute to PN are unknown. To identify specific lipid changes associated with PN, we conducted an untargeted lipidomics analysis comparing the effect of high-fat diet (HFD) feeding on lipids in the plasma, liver, and peripheral nerve from three strains of mice (BL6, BTBR, and BKS). HFD feeding triggered distinct strain- and tissue-specific lipid changes, which correlated with PN in BL6 mice versus less robust murine models of metabolic dysfunction and PN (BTBR and BKS mice). The BL6 mice showed significant changes in neutral lipids, phospholipids, lysophospholipids, and plasmalogens within the nerve. Sphingomyelin (SM) and lysophosphatidylethanolamine (LPE) were two lipid species that were unique to HFD BL6 sciatic nerve compared to other strains (BTBR and BKS). Plasma and liver lipids were significantly altered in all murine strains fed a HFD independent of PN status, suggesting that nerve-specific lipid changes contribute to PN pathogenesis. Many of the identified lipids affect mitochondrial function and mitochondrial bioenergetics, which were significantly impaired in *ex vivo* sural nerve and dorsal root ganglion sensory neurons. Collectively, our data show that consuming a HFD dysregulates the nerve lipidome and mitochondrial function, which may contribute to PN in prediabetes.

## Introduction

Peripheral neuropathy (PN) is a common and highly morbid complication of prediabetes and type 2 diabetes (T2D) (Feldman et al., 2019). PN presents as a distal to proximal loss of sensation in the extremities with pain as a frequent feature (Feldman et al., 2019). While the pathogenesis of PN is incompletely understood, impaired peripheral nervous system bioenergetics under conditions of excess energy substrate is a central characteristic of PN (Feldman et al., 2017). In parallel, recent clinical studies highlight components of the metabolic syndrome as PN risk factors (Callaghan et al., 2016a; Callaghan et al., 2016b), suggesting lipids, including triglycerides (TGs), contribute to peripheral nervous system energy overload (Wiggin et al., 2009; Andersen et al., 2018).

Murine models of diet-induced obesity develop features of prediabetes and PN like that seen in humans; however, the genetic background of each mouse strain affects the degree of metabolic dysfunction and type of nerve fibers affected (Montgomery et al., 2013). Large nerve fibers confer proprioceptive information related to position and movement whereas small afferent Aδ fibers and unmyelinated C-fibers are responsible for temperature, pain, and nociceptive sensations. Prediabetes and T2D PN result from a combination of large and small fiber dysfunction. We recently reported the effects of high-fat diet (HFD) feeding on three mouse strains (BL6, BTBR, and BKS). Mice on the BL6 background gained weight throughout the 36-weeks study and developed features of the metabolic syndrome as well as large and small fiber PN similar to what is observed in humans with prediabetes (Hinder et al., 2017). In contrast, HFD-fed BTBR mice developed large fiber PN only and gained weight at the same rate as standard diet (SD)-fed BTBR for the first 24 weeks of the study. The final strain of mice fed a HFD, the BKS mice, also developed large fiber PN only but required genetic manipulation of the leptin receptor to gain weight from study onset.

The goal of the current study was to assess the association between disruptions in lipid composition and PN metabolic risk factors and disease severity. Because lipid levels profoundly impact mitochondrial bioenergetics (Rumora et al., 2018), we postulated that distinct nerve lipid levels would associate with PN under varying conditions of metabolic dysfunction. We conducted untargeted lipidomics of nerve, liver and plasma from HFD-fed BL6, BTBR and BKS mice and observed distinct changes in nerve, liver and plasma lipids in all three strains. Unique changes in mitochondrial lipid levels were observed within the nerves of HFD-fed BL6 mice with PN, the only strain that developed both large and small nerve fiber dysfunction. Further evaluation of mitochondrial bioenergetics in *ex vivo* sural nerves and sensory dorsal root ganglion (DRG) neurons from BL6 animals showed impaired mitochondrial bioenergetics, suggesting a role for nerve-specific lipid signatures in the pathogenesis of PN.

## Materials and Methods

### Mouse Model Description

Mouse strains included i) BKS-*wt* (C57BLKS/J #000662, Jackson laboratory, Bar Harbor, ME), ii) B6-*wt* (C57BL/6J #000664, Jackson Laboratory), and iii) BTBR-*wt* (BTBR T+ Itpr3tf/J #002282, Jackson Laboratory)*.* Mice from each strain were randomly assigned to two groups at 4 weeks of age and fed either a standard diet (SD) (#D12450-B, 10% kcal fat, Research Diets, New Brunswick, NJ) or a 54% HFD (#05090701, 54% kcal fat from lard, Research Diets) for 32 weeks, leading to six groups of male mice with 12 mice/group (HFD BKS, SD BKS, HFD B6, SD B6, HFD BTBR, SD BTBR). The fatty acid composition of each diet is provided in Supplementary Table S1. At the study end at 36 weeks of age, sciatic nerve, footpads, plasma, and liver samples were collected. Terminal metabolic measurements included body weight, fasting blood glucose, glucose tolerance, and glycated hemoglobin, as well as terminal neuropathy measurements including assessments of sural and sciatic nerve conduction velocities (NCV), measures of large fiber function, and intraepidermal nerve fiber density (IENFD), a measure of small nerve fiber function, were evaluated at 36 weeks of age. Plasma insulin, cholesterol, and triglycerides were also measured by Mouse Metabolic Phenotyping Centers (MMPC; Vanderbilt University, Nashville, TN; University of Cincinnati, Cincinnati, OH). All metabolic and neuropathy measurements were reported previously (Hinder et al., 2017). Herein, we conducted a follow-up untargeted lipidomics analysis on sciatic nerve, plasma, and liver from each group of mice. Mice were housed in a pathogen-free environment and animal husbandry was conducted by the University of Michigan Unit for Laboratory Animal Medicine. Animal protocols followed Diabetic Complications Consortium Guidelines (https://www.diacomp.org/shared/protocols.aspx) and were approved by the University of Michigan University Committee on Use and Care of Animals.

### Untargeted Lipidomics Profiling

Four sciatic nerves from each group of mice were selected blindly and submitted to the Michigan Regional Comprehensive Metabolomics Resource Core (MRC2; www.mrc2.umich.edu) for untargeted lipidomics, which was conducted as described previously (Sas et al., 2018). Briefly, lipids were extracted from each sample (plasma, homogenized sciatic nerve, or homogenized liver) according to a modified Bligh-Dyer protocol. Purified lipids from samples and quality controls were analyzed by liquid chromatography-tandem mass spectrometry (LC-MS/MS). LipidBlast (http://fiehnlab.ucdavis.edu/projects/LipidBlast) was used to identify lipids and MultiQuant (SCIEX, Concord, Canada) was used for lipid quantification. A total of 967 lipid species were detected within the sciatic nerve (Positive - 579; Negative—388), 1,339 lipid species were detected in the liver (Positive- 799; Negative—540), and 956 lipid species were detected in the plasma (Positive - 603; Negative—353).

### Untargeted Lipidomics Data Preprocessing and Analysis

Missing values in the raw data were imputed with the K-nearest neighbor method and normalized to internal standards using the R package pamr with the function pamr. knnimpute (https://www.rdocumentation.org/packages/pamr/versions/1.55/topics/pamr.knnimpute) (Troyanskaya et al., 2001). Euclidian was used as the distance metric (Troyanskaya et al., 2001). At least one internal standard for each lipid class was included in the analysis (Supplementary Tables S2–S4). Lipid species with a coefficient of variation >30% were removed and then lipid species from positive and negative ion modes were merged into a single dataset. Lipids measured in both positive and negative modes were assigned an average value from both modes. Lipid species with an odd number of carbons were removed because odd-chain lipids are rarely synthesized in mammalian systems and are typically obtained from the diet or by gut microbiota (Venn-Watson et al., 2020; Ampong et al., 2022). We also did not identify any significant changes in branched lipids in this study. To summarize lipid levels per class, the total values of lipid species in each class were summed and then log_2_-transformed. Heatmaps were generated to visualize the profiling pattern of each lipid class across different tissue and genetic background groups. Pearson correlation coefficients were calculated for each shared lipid species between different tissues (O'Brien et al., 2020). Lipid heatmaps were not displayed in the figures if the HFD compared to the SD had no significant impact on tissue lipid levels in a particular strain of mice.

### Identifying Important Lipid Species

A *t*-test was performed for each lipid species to determine significant differences between the HFD and SD groups. Lipid species with a *p*-value < 0.05 were deemed significant differential lipids. Partial least squares-discriminant analysis (PLS-DA) was also performed with mixOmics package (Rohart et al., 2017), to identify lipid species that carry the greatest class-separating information, represented by the first latent variable (Brereton and Lloyd, 2014). Tenfold cross-validation was used to select the tuning parameter (the number of components) for PLS-DA with the minimal overall error rate. Once the optimal number of components was decided, the PLS-DA was refit to the full dataset to obtain the final model. Score plots were generated to illustrate the difference between HFD versus SD for each genetic background (BKS, BL6, BTBR). The variable importance in projection (VIP) score for each lipid species was calculated as a weighted sum of the squared correlations between the PLS-DA components and the original lipid species (Galindo-Prieto et al., 2014). Lipid species with a VIP score >1 were selected as the important species, which contribute highly to group separation (Cho et al., 2008). All the above analyses were performed using R v3.5 (https://www.R-project.org/).

### Mitochondrial Bioenergetics Analysis

*Ex vivo* mitochondrial bioenergetics analysis was conducted on whole sural nerve tissue and primary DRG neurons dissected from 20-week HFD- versus SD-fed BL6 mice using an XF24 Extracellular Flux Analyzer (Agilent Technologies, Santa Clara, CA, United States). Bioenergetic analysis was conducted 3–6 h post mortem for both primary DRG neuron cultures and whole sural nerve. Whole sural nerves were dissected from four mice/group, placed in optimized energetics media, and arranged on an islet capture screen (Pooya et al., 2014). For DRG neuron cultures, DRG were extracted, dissociated into a single-cell suspension, and plated on a laminin-coated Seahorse plate, as described previously (Rumora et al., 2018; Rumora et al., 2019a; Rumora et al., 2019b). Whole sural nerves were then challenged by sequential addition of mitochondrial drugs in the following order: i) 12.6 μM oligomycin, ii) 20 μM carbonyl cyanide-4-(trifluoromethoxy)phenyl-hydrazone (FCCP), and iii) 2 μM antimycin A. DRG neurons were challenged with the consecutive injection of i) 1.25 mM oligomycin, ii) 100 or 600 nM FCCP, and iii) 1 mM antimycin A. All bioenergetics measurements were recorded by the Seahorse XF analyzer and bioenergetics parameters were analyzed using mitochondrial drug response curves, as described previously (Rumora et al., 2018). Results were normalized to tissue weight and mitochondrial copy number (see below). Data analysis was conducted on GraphPad Prism using one-way ANOVA with a Tukey *post*-test for multiple comparisons, two-way ANOVA with Bonferroni *post*-test for multiple comparisons, or unpaired *t*-test (Festing and Altman, 2002).

### Mitochondrial Copy Number Analysis

The sural nerve mitochondrial copy number was evaluated in HFD- versus SD-fed BL6 mice, as previously described (Rumora et al., 2018). Briefly, DNA was isolated using the AllPrep DNA/RNA Mini Kit (Qiagen, Germantown, MD, United States) from the sural nerves, which were used for mitochondrial bioenergetics analysis. Quantitation of mitochondrial cytochrome b (*cytob*) and nuclear tyrosine 3-monooxygenase/tryptophan five- monooxygenase activation protein (*Ywhaz*) was evaluated using Power SYBR Green PCR Master Mix (Thermo Fisher Scientific) on a StepOnePlus Real-Time PCR system (Thermo Fisher Scientific), as described previously (Rumora et al., 2018). The standard curve method was used for *cytob* and *Ywhaz* gene quantitation.

## Results

### Tissue Lipidomics Profiling of HFD BL6, BTBR, and BKS Mice

**FIGURE 1:**
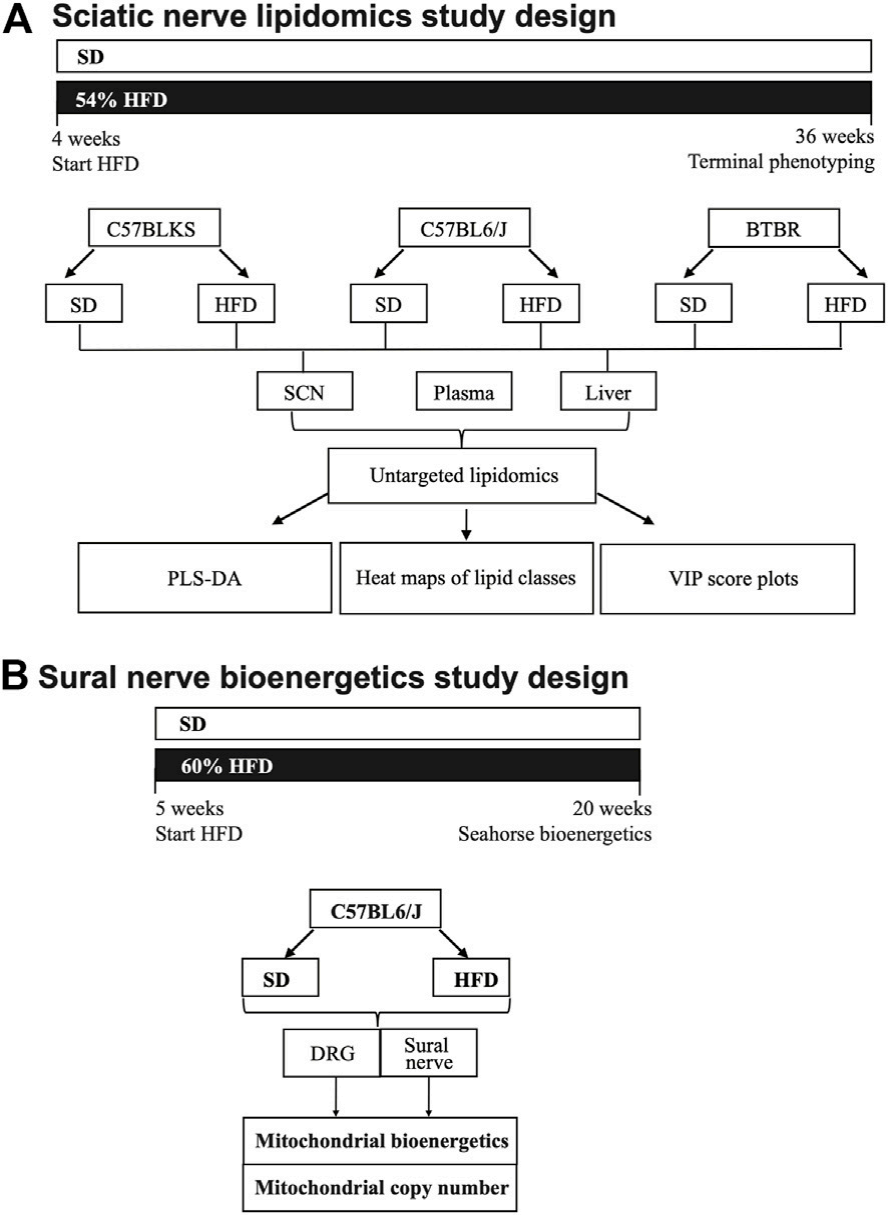
Study paradigm and workflow for lipidomics **(A)** and mitochondrial bioenergetics analysis **(B)**. **(A)** Three strains of mice (C57BLKS/J, C57BL6/J, and BTBR) were divided into two groups per strain and fed either a standard diet (SD) or 54% high-fat diet (HFD). Mice were fed SD or HFD for 32 weeks and phenotyped for metabolic and neuropathy parameters at 36 weeks. At 36 weeks, sciatic nerve, liver, and plasma were collected and processed for LC-MS/MS lipidomics analysis. Lipidomics data were subjected a bioinformatics pipeline including PLS-DA, heat maps, and VIP score plots. **(B)** DRG neuron and sural nerve mitochondrial bioenergetics were evaluated in BL6 mice fed a SD or 60% HFD from 5 to 20 weeks. At 20 weeks of age, mitochondrial bioenergetics and mitochondrial copy number were assessed in DRG neurons and whole sural nerves from HFD BL6 mice compared to SD BL6 mice.

**FIGURE 2:**
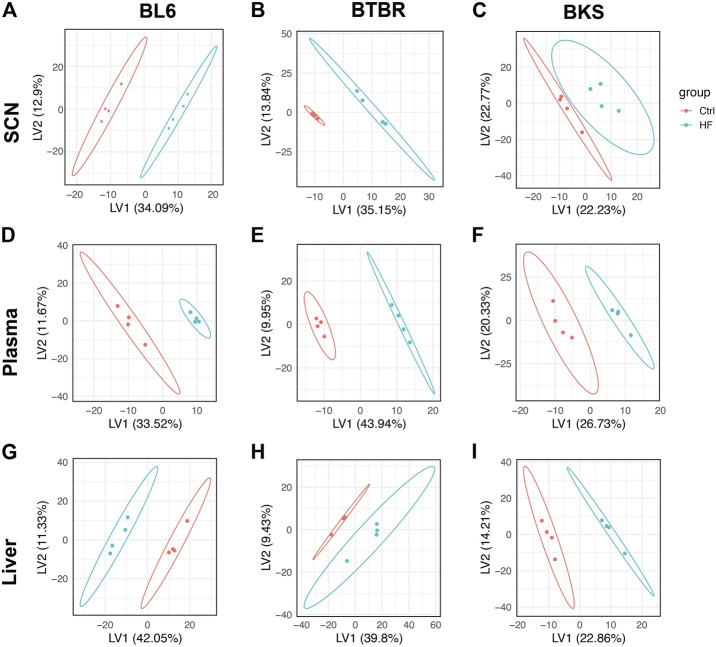
Score plots of lipid changes across species. Partial least squares-discriminant analysis (PLS-DA) showed strain-dependent separation of lipid species between the SD (red) and HFD mice (blue); dots represent individual mice. BL6 mice with weight gain, dyslipidemia, and both large fiber and small fiber PN **(A)** and BTBR mice with weight gain and large-fiber PN **(B)** showed distinct separation of sciatic nerve (SCN) lipids between SD and HFD groups compared to BKS mice with large fiber PN without weight gain **(C)**. Whereas, clear separation between plasma **(D–F)** and liver lipids **(G–I)** was visible across murine strains.

Untargeted lipidomics was performed on the sciatic nerve, plasma, and liver from BL6, BTBR, and BKS mice fed either SD or 54% HFD for 36 weeks (Figure 1A). PLS-DA score plots showed a clear separation between lipid species in the sciatic nerve of HFD BL6 mice with large and small fiber PN and HFD BTBR mice with large fiber PN, versus SD BL6 and SD BTBR mice without PN (Figures 2A,B). Sciatic nerve lipid profiles from HFD-fed BKS mice that had no weight gain compared to SD-fed animals show less separation between HFD and SD score plots (Figure 2C). Elevated plasma insulin levels and large fiber PN, based on slowed sciatic and sural nerve conduction velocities, were present in all strains. However, only BL6 mice fed a HFD had highly significant weight gain throughout the entire study (8-, 16-, 24-, 36- weeks) compared to SD-fed BL6 animals. At the study end, these animals also had statistically elevated cholesterol levels and low IENFDs, a marker of small fiber PN (Supplementary Table S5) (Hinder et al., 2017). HFD-fed BTBR mice gained weight at a similar rate as SD-fed animals for the first 24 weeks of the study, and only at the 36-weeks time point were significantly heavier than their SD-fed counterparts. These HFD animals had higher levels of fasting glucose than the SD-fed animals with no changes in lipid levels. In contrast both SD- and HFD-fed BKS mice gained weight at equivalent rates and had no evidence of elevated cholesterol or fasting glucose (Supplementary Table S5) (Hinder et al., 2017). The greater separation between the score plots of sciatic nerve lipids from HFD- vs SD-fed BL6 and BTBR mice shows that changes in nerve lipid composition are associated with distinct PN phenotypes and metabolic changes including weight gain, fasting glucose, and plasma insulin (Supplementary Table S5) (Hinder et al., 2017). Unlike the strain-dependent sciatic nerve lipid profiles, the liver and serum lipid profiles had a distinct separation between HFD versus SD groups, regardless of strain (Figures 2D–I). These results suggest that tissue-specific sciatic nerve lipid profiles are associated with distinct types of PN as defined by large and small nerve fiber involvement and metabolic dysfunction.

### Triglycerides and Diacylglycerols

**FIGURE 3:**
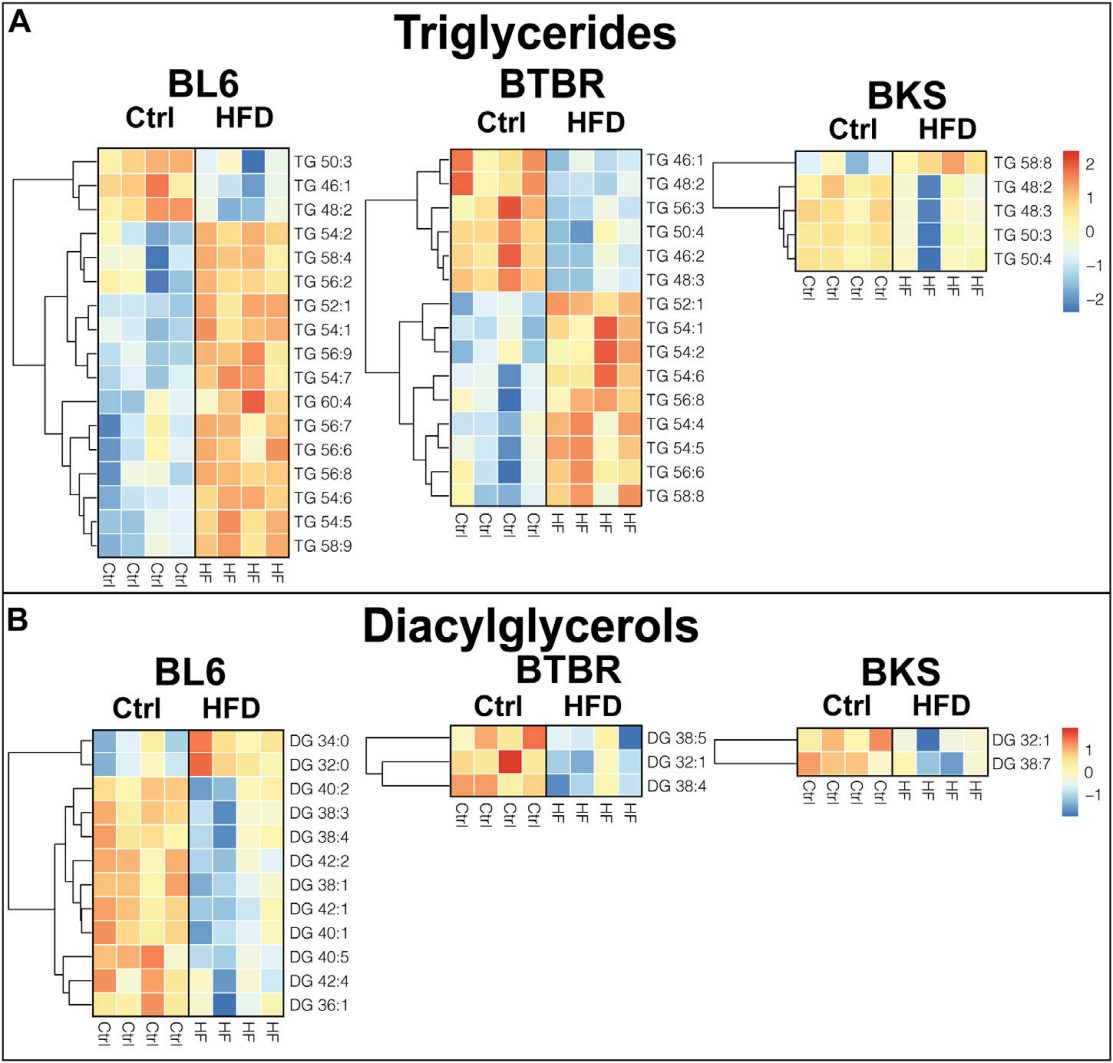
Heat maps of neutral lipids in the sciatic nerve of BL6, BTBR, and BKS mice fed the SD or HFD. **(A)** Sciatic nerve triglyceride (TG) chain length and degree of saturation were significantly altered in HFD sciatic nerve from BL6 and BTBR mice. **(B)** Sciatic nerve diacylglycerols (DGs) were significantly altered by the HFD in all three strains. *t*-test, *p*-value < 0.05.

To identify tissue-specific changes in lipid profiles that associate with PN, we generated heat maps of significantly altered (*p* < 0.05) lipid species in mice fed a HFD compared to SD. Out of a total of 57 detected TG species in the sciatic nerve, 17 were altered in BL6 mice, 15 in BTBR mice, and five in BKS with HFD feeding (Figure 3A). The chain length and saturation degree of sciatic nerve TGs were also altered by a HFD. Both BL6 and BTBR strains fed a HFD experienced weight gain by 36 weeks, developed at least two measures of metabolic dysfunction, and exhibited a higher abundance of sciatic nerve long-chain TGs, which contrasted with a higher level of shorter-chain TGs in BL6 and BTBR mice fed a SD. The highly abundant sciatic nerve long-chain TGs in HFD-fed BL6 and BTBR also showed a higher degree of acyl chain unsaturation. These HFD-induced changes in nerve TGs correlated with large fiber PN in both BL6 and BTBR mice. Conversely, sciatic nerve from BKS mice that did not gain weight and developed only one measure of metabolic dysfunction, showed changes in TG level but no distinct changes in TG chain length or saturation.

Diacylglycerols (DGs) were significantly altered in the sciatic nerve of all three strains of mice fed a HFD. A total of 35 DGs were analyzed in the sciatic nerve, of which 12 in BL6, three in BTBR, and two in BKS sciatic nerve were significantly affected by consuming a HFD (Figure 3B). Only sciatic nerve from BL6 mice with both large and small fiber PN showed changes in DG chain length, while BTBR and BKS mice had an overall decrease in DGs with HFD feeding compared to their respective SD controls. Interestingly, changes in chain length were opposite in DGs versus TGs, with BL6 sciatic nerve displaying greater shorter-chain and lower longer-chain DG levels in animals fed a HFD compared to animals on a SD. Collectively, these results suggest that elevated long-chain TGs and shorter-chain DGs in sciatic nerves correlate with weight gain, metabolic dysfunction, and large and small fiber PN in HFD BL6 mice after 36 weeks.

### Phospholipids

**FIGURE 4:**
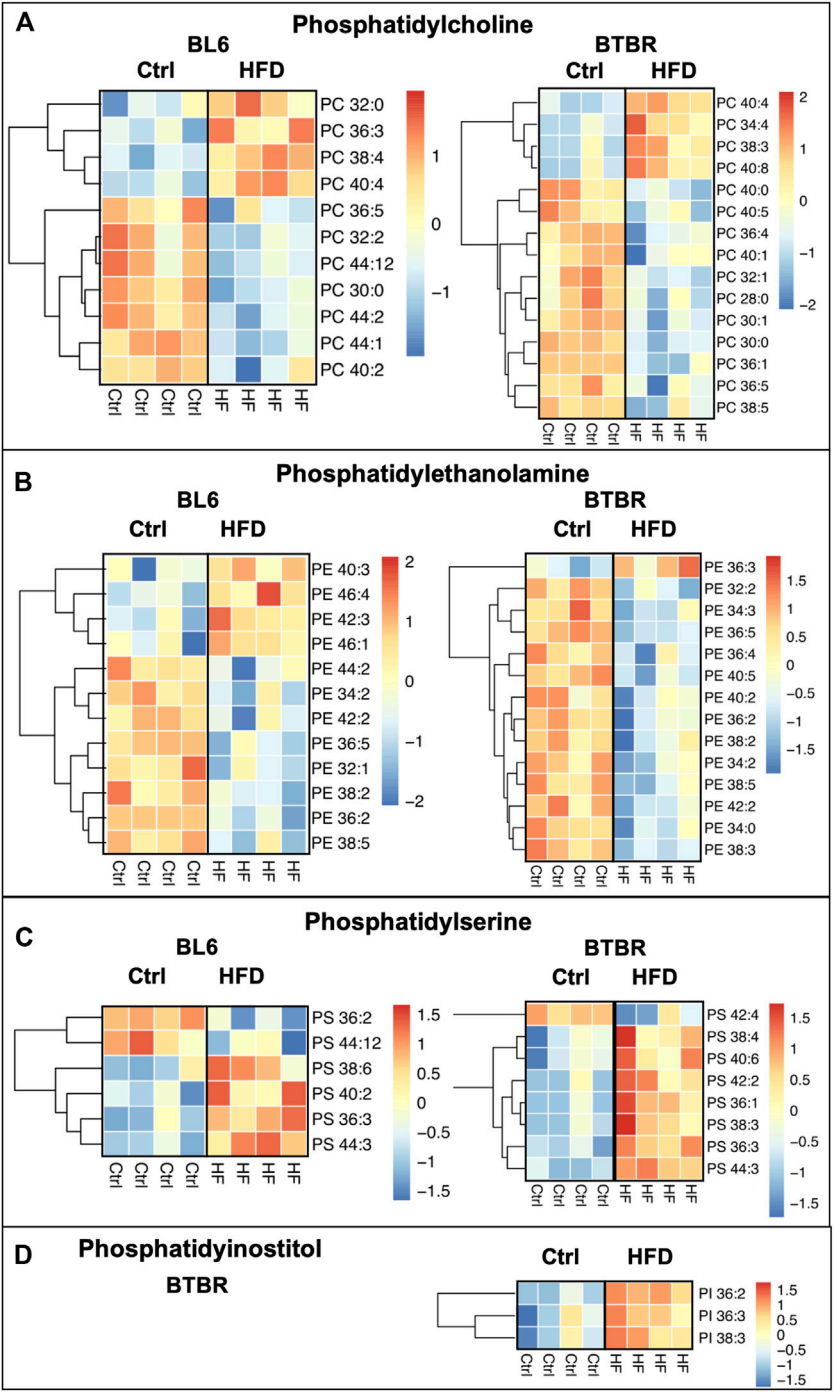
Heat maps of BL6, BTBR, and BKS sciatic nerve phospholipids. All phospholipid levels including **(A)** phosphatidylcholine (PC) **(B)** phosphatidylethanolamine (PE) **(C)** phosphatidylserine (PS) and **(D)** phosphatidylinsotitol (PI) were altered by the HFD in BL6 and BTBR mice. *t*-test, *p*-value < 0.05.

HFD feeding drastically dysregulated phospholipids in the sciatic nerve of BL6 models with large and small fiber PN and BTBR models with large fiber PN, which both experienced weight gain and metabolic dysfunction (Figure 4). However, phospholipids were unaffected in the sciatic nerve of HFD-fed BKS mice, the strain that did not gain weight and developed less metabolic dysfunction with a HFD. Collectively, these findings suggest a role for phospholipids in large fiber PN pathogenesis associated with metabolic dysfunction. Within the sciatic nerve, BL6 mice had a higher level of short-chain phosphatidylcholines (PCs) and long-chain phosphatidylethanolamines (PEs) with HFD feeding versus SD mice (Figures 4A,B). Although the levels of certain PC and PE species were also affected in HFD-fed BTBR sciatic nerve, there were no distinct changes in the chain length. In both BKS and BTBR mice fed a HFD, levels of specific phosphatidylserine (PS) species were altered, but without discernable changes in chain length (Figure 4C). Decreases in phosphatidylinositol (PI) species were exclusive to HFD-fed BTBR sciatic nerve (Figure 4D).

**FIGURE 5:**
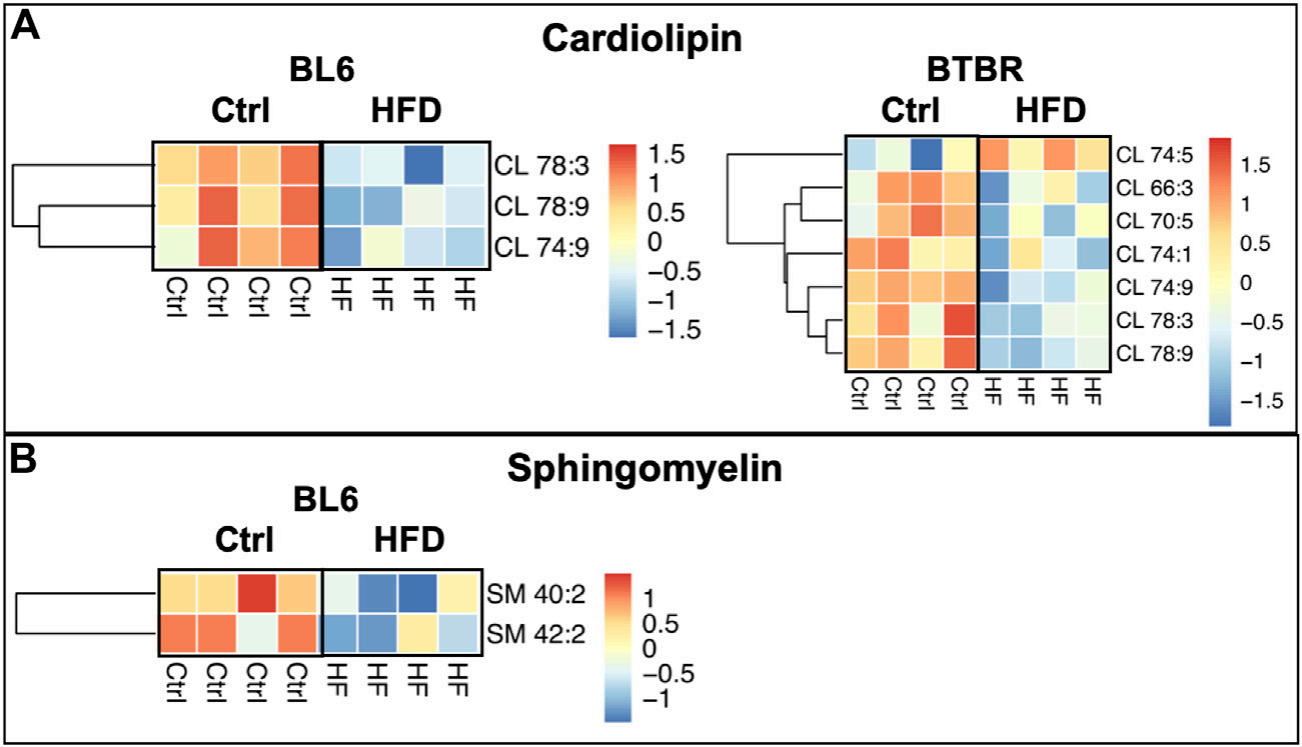
Heat maps of BL6 and BTBR sciatic nerve cardiolipin (CL) and sphingomyelin (SM). HFD BL6 and BTBR mice show global decreases across all **(A)** CL and **(B)** SM lipid species. *t*-test, *p*-value < 0.05.

Levels of mitochondrial phospholipid cardiolipin (CL) were significantly reduced within the sciatic nerves of BL6 and BTBR mice after HFD feeding whereas sphingomyelin (SM) levels were only reduced in the sciatic nerves from HFD-fed BL6 mice (Figures 5A,B). Conversely, a HFD did not affect SM and CL levels in the sciatic nerves of BKS mice. Since CL and SM were only reduced in sciatic nerve from animals that gained weight and were metabolically dysfunctional, these phospholipids may play an important role in PN pathogenesis associated with metabolic dysfunction.

### Lysophospholipids and Plasmalogens

**FIGURE 6:**
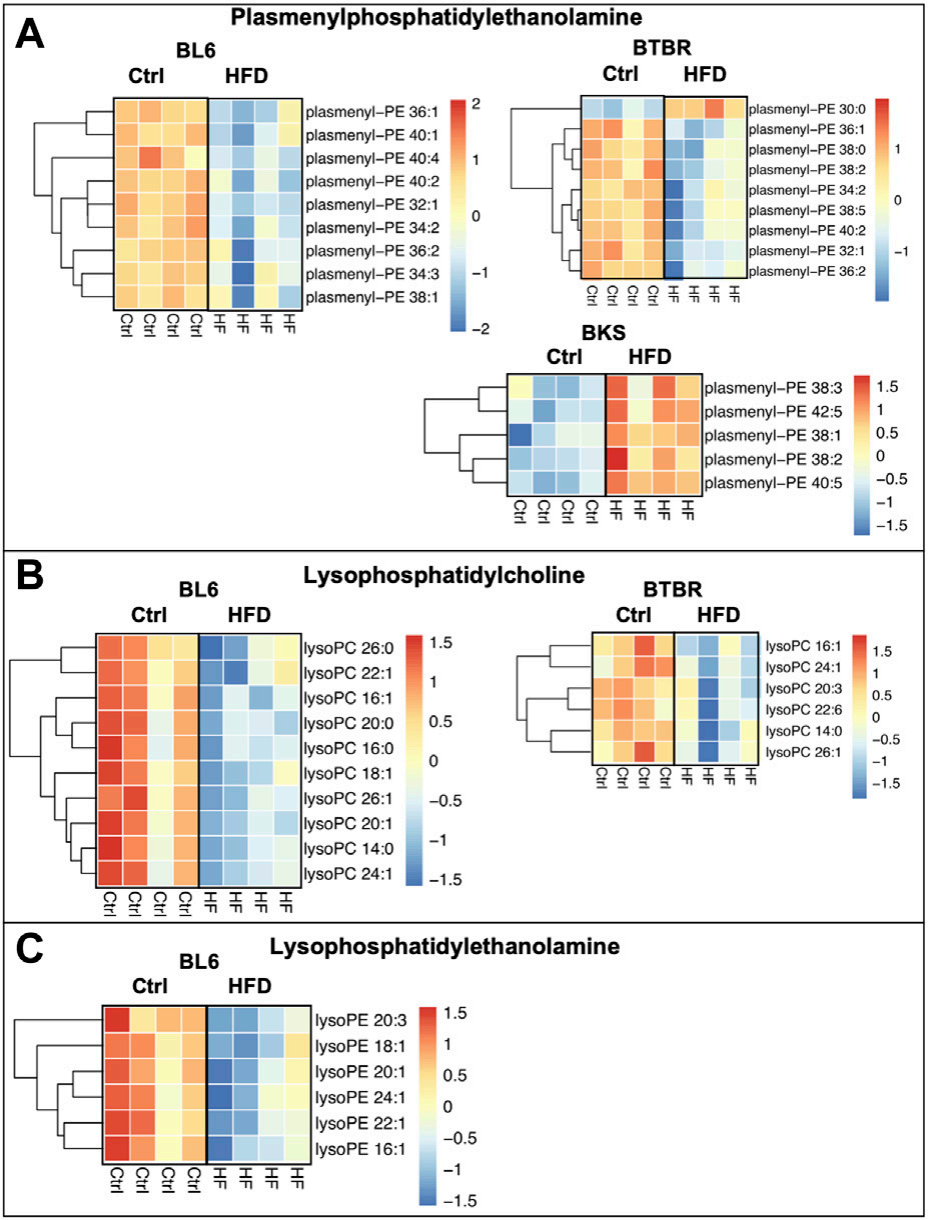
Heat maps of BL6, BTBR, and BKS sciatic nerve plasmalogens and lysophospholipids. **(A)** HFD BL6 and BTBR mice show a global decrease in plasmenyl-phosphatidylethanolamine (plasmenyl-PE) whereas BKS mice have an increase in plasmenyl-PE. **(B)** Lysophosphatidylcholine (LPC) species were also significantly decreased in HFD BL6 and BTBR mice. **(C)** LPE species were decreased exclusively in the sciatic nerve of HFD BL6 mice only. *t*-test, *p*-value < 0.05.

The HFD feeding significantly altered lysophospholipid and plasmalogen lipids in the sciatic nerve for all three strains of mice when compared to SD (Figures 6A–C). Lysophosphatidylcholine (LPC) and plasmenyl-phosphatidylethanolamine (plasmenyl-PE) lipid species were significantly decreased in the sciatic nerves of BL6 and BTBR mice fed a HFD compared to SD. The HFD-fed BKS mice with no metabolic dysfunction displayed a significant decrease in LPC and an increase in plasmenyl-PE. A reduction in lysophosphatidylethanolamine (LPE) in the sciatic nerve occurred only in BL6 mice fed a HFD, and not the two other strains, suggesting an association with LPE and both large and small fiber PN in the murine model that most closely replicates the human condition.

### Liver and Plasma Lipid Profiles

The sciatic nerve lipidome is modulated by changes in plasma lipid levels, whereas the liver is a major regulator of circulating plasma lipid levels (O'Brien et al., 2017; O'Brien et al., 2020). Therefore, we compared the liver and plasma lipid levels across all murine strains with HFD versus SD feeding and found major changes in lipid profiles regardless of murine strain. Although no significant differences in plasma TG level were detected in all murine strains (Supplementary Table S5), the chain length of plasma TGs changed with HFD feeding. The BL6 and BTBR mice, but not the BKS animals, had significant elevations in long-chain TGs in plasma after HFD feeding compared to SD groups (Supplementary Figure S1). The levels of several plasma DG and cholesterol ester species were uniquely altered in HFD BTBR mice after HFD feeding (Supplemental Figure 1B-C). Plasma phospholipid levels were changed across all murine strains fed the HFD but showed no significant difference in chain length or degree of saturation (Supplementary Figure S2). Interestingly, plasma SM levels were elevated in all HFD-fed murine strains including HFD BL6 mice, which contrasted with decreased SM levels in the HFD BL6 sciatic nerve (Supplementary Figure S3). The levels of plasma plasmenyl-PE, plasmenylphosphatidylcholine (plasmenyl-PC), LPE, and LPC were also significantly upregulated or downregulated by the HFD feeding depending on the murine strain (Supplementary Figure S4).

The liver had distinct changes in neutral lipids including significant increases in TG and DG chain length, as well as an overall decrease in DGs, in BL6 fed a HFD compared to a SD diet (Supplementary Figure S5). Conversely, the levels of liver TGs were significantly decreased in BTBR and BKS mice fed a HFD (Supplementary Figure S5). Phospholipids were also significantly altered in the liver of all three mouse models. The level of PCs and PEs were significantly decreased in the liver of all three murine strains with HFD feeding (Supplementary Figure S6). Other phospholipid groups including PI, PS, phosphatidylglycerol, and phosphatidic acid were also decreased in a strain-dependent manner in the liver of these mice (Supplementary Figure S6). As in the sciatic nerve, there was a significant decrease in CL, plasmenyl-PC, LPC, and LPE in the liver of BL6 mice fed a HFD (Supplementary Figures S7A, S8A–D). The levels of specific species of SM, plasmalogens, and lysophospholipids were altered in certain strains of HFD mice [SM (BL6, BKS), plasmalogens (BL6, BTBR, BKS), and lysophospholipids (BL6, BTBR)], but there were no distinct changes in chain length or degree of saturation.

### Top Lipids Contributing to Peripheral Neuropathy in BL6 HFD-Fed Animals

**FIGURE 7:**
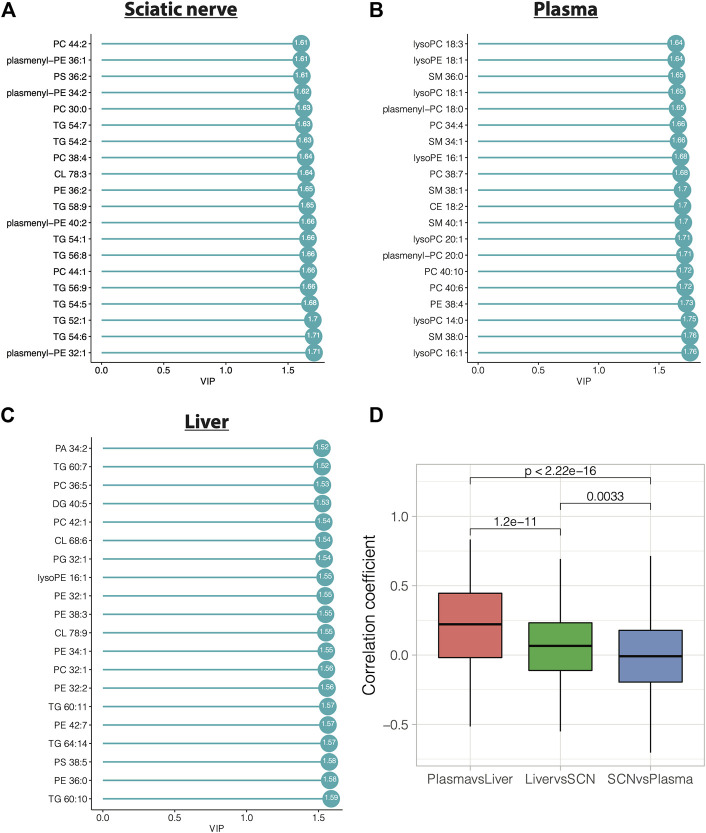
Variable importance in projection (VIP) score plots of the top 20 PLS-DA lipids in **(A)** sciatic nerve, **(B)** plasma, and **(C)** liver, that separate HFD BL6 mice from SD mice. **(D)** Pearson correlation coefficients for each shared lipid species between plasma vs liver, liver vs sciatic nerve, and sciatic nerve vs plasma.

We have previously reported that HFD feeding of BL6 mice leads to metabolic dysfunction and large and small fiber PN that most closely resembles that seen in humans (Hinder et al., 2017; Rumora et al., 2019b; O'Brien et al., 2020). To determine lipids most significantly linked to pathogenesis of both large and small fiber PN, we identified the lipid species in sciatic nerves, plasma, and liver that contributed the most to diet-induced group separation among BL6 animals by VIP plots and correlation coefficient analysis. A total of 166 sciatic nerve lipids, 141 plasma lipids, and 240 liver lipids had VIP values greater than 1. The top 20 lipids with the highest VIP values were 9 TGs, four PCs, four plasmenyl-PEs, one PS, one PE, and one CL species, which were significantly altered in sciatic nerves (Figures 7A–C). Lipid VIP scores for each tissue are provided in Supplementary Tables S6–S8. Plasma lipids were also significantly impacted by HFD feeding including five LPCs, five SMs, three PCs, two plasmenyl-PCs, two LPEs, one PE, one CL species, and one cholesterol ester. Important liver lipids affected by HFD feeding included six PEs, 4 TGs, three PCs, two CLs, one LPE, one PS, one phosphatidic acid, one DG, and one phosphatidylglycerol species. We next assessed lipid correlations across tissues and found, among the 35 differentially altered lipids, plasma and liver had greater overlap in shared lipids versus sciatic nerve (Figure 7D). Finally, we directly compared the liver, plasma, and sciatic nerve lipid levels in BL6 mice. Interestingly, lipid levels in the sciatic nerve were distinct from lipid levels in the plasma or liver in BL6 mice fed the SD and the HFD (Supplementary Figures S9A,B).

To identify lipid changes that contribute to PN in the different mouse strains, we compared sciatic nerve lipids with VIP >1 across the three strains of mice. We identified 33 shared lipid changes between all murine strains, 57 shared lipid changes between sciatic nerve from BL6 and BTBR mice, and 20 shared lipid changes in sciatic nerve from BL6 and BKS mice (Supplementary Figures S10 and Supplementary Table S9). All HFD murine strains developed large fiber neuropathy and had changes in the level of neutral lipids (triglycerides and diacylglycerols) indicating that changes in neutral lipid species may contribute to large nerve fiber damage. HFD BL6 and BTBR mice that developed large fiber neuropathy associated with metabolic dysfunction shared many lipid changes in lysophospholipids and plasmalogens that were less distinct in HFD BKS mice, indicating that these lipid species may contribute to large fiber neuropathy in metabolic dysfunction.

### HFD Impairs Mitochondrial Bioenergetics Within DRG Neurons and the Sural Sensory Nerve

**FIGURE 8:**
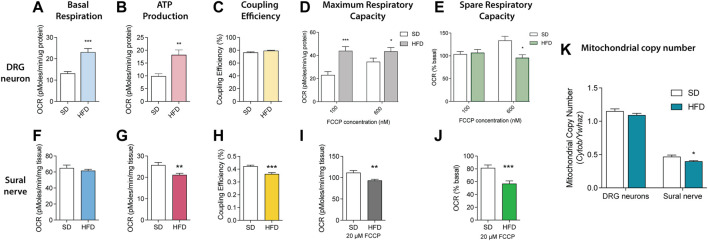
Mitochondrial bioenergetics in SD vs HFD DRG neurons and sural nerves. Resting bioenergetics parameters including **(A)** basal respiration and **(B)** ATP production were significantly increased in DRG neurons from HFD mice compared to SD mice, whereas **(C)** coupling efficiency was unaffected. **(D)** HFD DRG neurons challenged with 100 and 600 nM FCCP had increased maximum respiratory capacity, but significantly diminished spare respiratory capacity with 600 nM FCCP **(E)** relative to SD DRG neurons. The HFD had no effect on sural nerve **(F)** basal respiration but significantly reduced both **(G)** ATP production and **(H)** coupling efficiency compared to the SD sural nerve. Challenging the HFD sural nerve with 20 μM FCCP significantly impaired both **(I)** maximum respiratory capacity and **(J)** spare respiratory capacity compared to SD sural nerves. **(K)** Mitochondrial copy number was reduced in HFD sural nerve. Data mean ± SEM, n = 15 mice/group sural nerves; *p < 0.05, **p < 0.01, ***p < 0.001.

Since essential mitochondrial phospholipids, including PE, PC, PI, PS, and CL, were significantly altered in sciatic nerves of BL6 mice fed a HFD, we next evaluated the impact of HFD on *ex vivo* mitochondrial function. Mitochondrial bioenergetic analyses were performed on the DRG sensory neurons and the sural sensory nerve. DRG neurons showed significant increases in basal respiration and ATP production with no discernable change in coupling efficiency at rest (Figures 8A–C). DRG neurons from HFD-fed BL6 mice challenged with both 100 and 600 nM FCCP had significantly higher maximum spare respiratory capacity relative to the BL6 DRG neurons from SD, but loss of spare respiratory capacity at 600 nM FCCP (Figures 8D,E). Basal ATP production and coupling efficiency were significantly reduced in BL6 sural nerves from HFD-fed animals, while the basal respiration was not impacted, compared to sural nerves from animals fed a SD (Figures 8F–H). Sural nerve mitochondria also showed a significant decrease in maximum respiratory capacity and spare respiratory capacity in HFD-fed versus SD animals (Figures 8I,J). Mitochondrial copy number was also significantly lower with HFD feeding in sural nerves but not DRG neurons (Figure 8K). These results indicate that a HFD induces DRG and sural nerve mitochondrial dysfunction, correlating with altered mitochondrial lipid levels, which may contribute to the loss of sensory nerve function in PN.

## Discussion

Multiple clinical studies identify components of the metabolic syndrome, including dyslipidemia and elevated TGs, as important PN risk factors (Wiggin et al., 2009; Andersen et al., 2018). Preclinical research shows these same risk factors adversely impact axonal mitochondrial trafficking and bioenergetics (Rumora et al., 2018; Rumora et al., 2019a; Sajic et al., 2021) resulting in bioenergetic failure in distal peripheral nerve axons and PN (Feldman et al., 2017). Despite the relevance of lipids, both as PN risk factors and in PN pathogenesis, the precise circulating and nerve lipid species most important to PN remain unknown. Importantly, the correlation of plasma and liver lipidome to nerve lipidome is also incompletely understood, despite the fact that a circulating lipidomic signature correlated to that identified in the nerve could serve as a disease biomarker. Thus, we undertook a systematic study of sciatic nerve, plasma, and liver lipidomics of three HFD-fed mouse models of varying metabolic and neuropathic phenotypes. The first model, HFD-fed BL6 mice, gain weight and develop insulin resistance, dyslipidemia and both large and small fiber PN, metabolic and PN features similar to those reported in humans with prediabetes (Hinder et al., 2017). The second model, BTBR mice, are resistant to weight gain until 36 weeks but develop insulin resistance, hyperglycemia and large fiber PN. Lastly, the third model, BKS mice fed a HFD diet develop only insulin resistance and large fiber PN without gaining weight. Although large fiber PN was detected in all strains of mice by 36 weeks of age, only BL6 mice consistently developed the diet-induced metabolic dysfunction and sensory PN that closely mimics the human condition. We, therefore, reasoned that comparing the lipid signatures in these different strains with varying metabolic and neuropathic phenotypes would identify tissue-specific lipids important in PN pathogenesis.

We found that score plots of sciatic nerve lipids separated BL6 and BTBR mice fed a HFD from their SD counterparts, aligning with the presence of weight gain and large fiber PN at 36 weeks in these HFD-fed strains. In contrast, BKS animals did not experience this same diet-mediated separation in sciatic nerve lipids and in parallel did not gain weight with a HFD. These data show that nerve-specific lipid changes correlate with weight gain from HFD feeding and support the idea that diet-induced lipid changes impact tissue function, especially in tissues with diverse lipid composition, such as the peripheral nervous system (Surma et al., 2021). We also discovered distinct changes in the sciatic nerve lipidome of HFD-fed BL6 and BTBR mice with large fiber PN, including neutral lipids (TGs, DGs), phospholipids, lysophospholipids, and plasmalogens. However, changes in nerve SMs and LPE levels were unique to HFD-fed BL6 mice who robustly model large and small fiber PN and are the only strain to develop plasma dyslipidemia. These nerve lipid classes may selectively contribute to small fiber nerve damage commonly associated with obesity, the metabolic syndrome, and prediabetes (Palavicini et al., 2020). Liver and plasma lipid profiles, that presumably dictate the sciatic nerve lipidome, also changed significantly in response to a HFD in all mouse strains. Since none of these plasma or liver changes were specific to animals with varying degrees of PN and metabolic dysfunction, it suggests that nerve-specific lipid changes specifically contribute to PN. Many of the nerve lipids identified in BL6 mice fed a HFD are critical for mitochondrial function; indeed, sural nerves from BL6 mice fed a 60% HFD were characterized by a loss of respiratory capacity in both the basal resting and energetically challenged states. Collectively, these results indicate that changes in the peripheral nerve lipidome associate with specific PN phenotypes (large and/or small fiber dysfunction) and likely contribute to mitochondrial dysfunction in PN.

An accumulation of long-chain TGs and short-chain DGs in the sciatic nerve was associated with PN in HFD BL6 mice with small and large fiber PN and in BTBR mice with large fiber PN, indicating that long-chain fatty acids from the diet are incorporated into TGs, mobilized into the plasma, and integrated into the sciatic nerve lipidome (Tracey et al., 2018). These results are consistent with previous studies showing elevated TGs in the sciatic nerve of neuropathic BL6 mice fed 60% HFD (O'Brien et al., 2020). In fact, gene expression of diacylglycerol acyltransferase 2 (DGAT2), the rate-limiting enzyme for TG biosynthesis was significantly increased in the sciatic nerve of these neuropathic BL6 mice fed 60% HFD. DGAT2 was also elevated in sural sensory nerve biopsies from T2D humans with PN, suggesting that nerve TG synthesis is elevated in PN (O'Brien et al., 2020). In the current study, TGs also displayed longer hydrocarbon chains and a greater degree of unsaturation in the HFD BL6 sciatic nerve, consistent with findings in plasma of type 2 diabetic human subjects with dyslipidemia and progressively worsening diabetic complications (Afshinnia et al., 2018; Afshinnia et al., 2019). The increase in TG hydrocarbon chain unsaturation in the sciatic nerve might be a compensatory mechanism to replace saturated TG hydrocarbon chains with polyunsaturated hydrocarbon chains in an attempt to prevent nerve lipid peroxidation (Bailey et al., 2015; Ackerman et al., 2018). Alternatively, the observed mobilization of long-chain saturated fatty acids from TGs can uncouple the mitochondrial membrane and impair mitochondrial oxidative phosphorylation, which could have contributed to the observed nerve injury (Murray et al., 2011).

In contrast to TGs, saturated DGs, including DGs 30:0–34:0, were significantly elevated in the sciatic nerve of HFD BL6 mice. The predominant DG species in standard rodent sciatic nerve are unsaturated DGs 38:4 (18:0/20:4) and 34:1 (16:0/18:1) (Eichberg and Zhu, 1992). Therefore, our findings indicate that HFD consumption triggers the incorporation of saturated fatty acids, such as palmitic acid and stearic acid, into sciatic nerve DGs in HFD BL6 mice with small and large fiber PN and dyslipidemia. This accumulation of saturated DGs in the sciatic nerve may underlie nerve damage by mediating lipotoxicity, mitochondrial dysfunction, endoplasmic reticulum stress, or apoptosis (Akoumi et al., 2017).

Our findings suggest that redirecting lipid biosynthetic pathways away from TG/DG synthesis could provide a viable therapeutic approach to treating PN. In support of this idea, inhibiting DGAT and lipin1, a DG synthesizing enzyme, promotes axon regeneration in peripheral neurons by reducing TG/DG synthesis and stimulating phospholipid synthesis (Yang et al., 2020). Furthermore, modulating DG levels in the sciatic nerve of STZ-treated rats confers neuroprotection and improves PN measures (Wang et al., 2021). Future preclinical studies focused on nerve-specific TG/DG biology in the setting of dyslipidemia and metabolic dysfunction could facilitate the development of targeted interventions for the treatment of PN.

Phospholipids, including PE, PC, PS, and CL, were significantly altered in the sciatic nerve of both HFD-fed BL6 and BTBR mice but not BKS mice, indicating a major shift in the nerve phospholipid content in response to HFD feeding that correlates with weight gain. These results parallel previous studies in murine models and humans with metabolic syndrome, prediabetes, and T2D (O'Brien et al., 2020; Rumora et al., 2021), suggesting that changes in nerve phospholipids contribute to large fiber PN pathogenesis in these disease states. Phospholipids make up approximately 57% of lipids in the cell bodies and axons of peripheral neurons and 40% of lipids in the myelin sheath (Calderon et al., 1995; Poitelon et al., 2020; Hornemann, 2021). Alterations in phospholipid levels can trigger aberrant changes in cellular signaling (Nishizuka, 1992), cell membrane structure (Kuge et al., 2014), and membrane dynamics in neurons (Tracey et al., 2018). Importantly, phospholipids are a major constituent of the mitochondrial membrane and play an integral role in regulating mitochondrial function.

The most abundant phospholipids in the inner mitochondrial membrane (PE, PC, CL) (Basu Ball et al., 2018) were those most changed in HFD BL6 sciatic nerve in this study, supporting the idea that these phospholipid changes could alter mitochondrial bioenergetics (Schenkel and Bakovic, 2014). Changes in the levels of inner mitochondrial membrane PE, PC, and CL result in the improper assembly of the mitochondrial electron transport supercomplexes, impairing oxidative phosphorylation (Tasseva et al., 2013). Changes in CL are of particular interest since CL is exclusively found in mitochondria and modulates the assembly of respiratory chain supercomplexes III and IV (Zhang et al., 2005), mitochondrial membrane potential (Ghosh et al., 2020), mitochondrial bioenergetics (Paradies et al., 2014), reactive oxygen species production (Falabella et al., 2021), and apoptotic signaling and mitochondrial dynamics (Falabella et al., 2021). The shift in PS and PE lipids, as well as the loss of CL, within the sciatic nerves of HFD BL6 mice may destabilize mitochondrial respiratory chain complexes, thereby reducing the efficiency of oxidative phosphorylation and injuring the peripheral nerves.

The most distinct lipid change was a global decrease in lysophospholipids (LPC, LPE) and plasmenyl-PE in the sciatic nerve of HFD BL6 and BTBR mice compared to BKS mice. A decrease in lysophospholipids was recently reported in both sciatic nerve (O'Brien et al., 2020) and plasma (Guo et al., 2021) from mice and humans, respectively, with PN and metabolic disease. Elevated levels of LPC are also implicated in neuropathic pain associated with chemotherapy-induced neuropathy (Rimola et al., 2020) and other painful neuropathies (Inoue et al., 2008). Lysophospholipids are generated from the hydrolysis of phospholipids (Tan et al., 2020), leading to elevated nitric oxide levels, which may damage peripheral nerves (Wang et al., 2013). Interestingly, LPCs and LPEs, were only decreased in HFD-fed BL6 and HFD-fed BTBR sciatic nerves emphasizing the possibility that sciatic nerve lysophospholipid levels may be strain-dependent (Hinder et al., 2017) and may contribute to the observed differences in PN among the three strains. The HFD BL6 sciatic nerve had the highest number of altered LPC species and was the only strain with HFD-induced alterations in LPE species indicating that altered lysophospholipids levels may contribute to small and large fiber PN associated with metabolic dysfunction. LPC levels are reportedly increased during painful PN (Wang et al., 2013), indicating that a loss of sensory function may be associated with the distinct decrease in LPC species in HFD-fed BL6 mice. Plasmenyl-PE is a plasmalogen, a family of lipids that contain arachidonic acid, a known mediator of nervous system lipid-signaling pathways (Murphy, 2017), membrane trafficking, and inflammatory pathways (Tracey et al., 2018). Critical for the formation of membrane rafts in the nervous system (Poitelon et al., 2020), loss of plasmalogen plasmenyl-PE in peripheral nerves may alter the lipid composition of myelin and ultimately lead to nerve damage.

Only two lipid species, SM and LPE, were dysregulated exclusively in the sciatic nerve from HFD BL6 mice with weight gain, dyslipidemia, and small and large fiber PN that mimics the human condition, compared to sciatic nerve from BTBR or BKS mice. Both SM and LPE were significantly decreased, indicating that the loss of SM and LPE within the sciatic nerve may contribute to small fiber damage within the nerve. This is supported by reports showing decreased plasma SM levels in patients with T2D (Rumora et al., 2021) and obesity (Guo et al., 2021). SM is an important nerve lipid of the myelin sheath, which protects and supports sensory nerve fibers (Poitelon et al., 2020; Hornemann, 2021). Although the role of LPE in the peripheral nervous system is less studied, changes in LPE levels are reported in other neurological disorders, including Alzheimer’s disease (Liu et al., 2021; Llano et al., 2021), emphasizing the importance of this lipid for proper nervous system function.

In the current study, HFD significantly impacted the liver and plasma lipid profile in all three murine strains, irrespective of PN. Since the peripheral nerves rely on both *de novo* lipogenesis and lipid uptake from circulation (Tracey et al., 2018; Poitelon et al., 2020), the saturation and chain length of circulating dietary fatty acids and complex lipids can influence the nerve lipidome. We have shown switching mice from a saturated fatty acid-rich HFD to a monounsaturated fatty acid-rich HFD rich significantly improves nerve function (Rumora et al., 2019b), likely because monounsaturated fatty acids restore mitochondrial function following saturated fatty acid-induced mitochondrial dysfunction (Rumora et al., 2018; Rumora et al., 2019a; Rumora et al., 2019b). Previous studies also describe elevated plasma and liver TGs in HFD-fed BL6 mice with PN, consistent with reports showing higher TGs in plasma of dyslipidemic rodents (Lupachyk et al., 2012), and plasma of diabetic (Wiggin et al., 2009; Callaghan et al., 2011; Smith and Singleton, 2013) and obese subjects with PN (Guo et al., 2021). However, we observed no strain-dependent differences in plasma or liver lipid classes that were unique to the HFD-fed BL6 mice or BTBR HFD-fed animals. These data suggest that nerve-specific lipid changes are a more important driver of PN pathogenesis than plasma or liver lipid signatures. In support of this idea, a recent study showed that statins alter circulating lipid levels in a T2D patient cohort from the ADDITION-Denmark study but have no effect on PN (Kristensen et al., 2020).

Lipids profoundly influence mitochondrial bioenergetics (Hinder et al., 2012; Aon et al., 2014); therefore, we determined whether changes in the peripheral nerve lipidome correlate with mitochondrial function distally in the *ex vivo* sural sensory nerve and proximally in sensory DRG neurons from HFD-fed BL6 mice. Although untargeted lipidomics was conducted on sciatic nerves to provide sufficient tissue for the lipidomic analysis, prediabetic and T2D PN is primarily a sensory neuropathy (Feldman et al., 2017), so mitochondrial bioenergetic analyses were performed on the sural sensory nerve and DRG sensory neurons. Since HFD-fed BL6 mice robustly mimic PN in humans with metabolic dysfunction, we postulated that *ex vivo* sural nerve and DRG neurons from HFD-fed BL6 mice would model changes in mitochondrial function that underlie diet-induced small and large fiber PN pathogenesis. Basal ATP production, coupling efficiency, and mitochondrial copy number were reduced in the sural nerves from HFD-fed animals, suggesting that mitochondrial energy production is compromised due to uncoupling of ATP production from mitochondrial respiration, as well as fewer mitochondria (Chowdhury et al., 2013). Challenging these sural nerves with mitochondrial uncoupler, FCCP, revealed a decrease in both maximum respiratory capacity and spare respiratory capacity, indicating the inability to increase ATP production to match increased energy demand.

In contrast, DRG neurons cultured from HFD-fed BL6 mice had significant increases in basal respiration and ATP production under resting conditions with no changes in coupling efficiency (Rumora et al., 2018), suggesting that mild uncoupling doesn’t occur despite the increase in basal respiration. The lack of uncoupling could in part prevent the formation of reactive oxygen species. The loss of spare respiratory capacity suggests the DRG neuron mitochondria are already functioning at maximum capacity and cannot increase energy output to meet increased energy demands. Elevated ATP production in DRG neurons may therefore be a compensatory mechanism to increase mitochondrial content and mitochondrial-derived ATP distally in the sural nerve, to maintain at least partial nerve function (Feldman et al., 2017).

Our study had several limitations. First, we used two different HFD paradigms including a 54% HFD for lipidomics studies versus a 60% HFD for mitochondrial bioenergetics. Since we previously showed lipid changes in the sciatic nerve of mice fed a 60% HFD by 16 weeks of age were similar to mice fed the 54% HFD at 36 weeks of age (O'Brien et al., 2020), we postulated that changes in mitochondrial bioenergetics would be similar across the two HFD paradigms. Future studies will test the effect of the two different HFD paradigms on mitochondrial bioenergetics in whole sural nerve and DRG neurons. Second, we were unable to determine the acyl chain composition of lipids in the untargeted lipidomics analysis. Targeted lipidomics platforms will be used in future studies to identify structural changes in sciatic nerve, liver, and plasma lipid species. It will be interesting to determine whether HFD impacts the identity of the acyl chains of key sciatic nerve lipid species we identified in this study. Further studies are also needed to determine the significance of odd chain lipids in HFD fed mice. Additionally, we will conduct transcriptomics on nerve, liver, and plasma from each mouse strain to assess changes in genes related to *de novo* lipogenesis and other metabolic pathways in each tissue. A third limitation of this study is the use of the sciatic nerve for untargeted lipidomics versus DRG neurons and sural nerve for mitochondrial bioenergetics analysis. Future directions will use targeted lipdomics or MALDI-MSI to correlate changes in mitochondrial bioenergetics function with lipid changes in DRG neurons and sural nerve. A fourth limitation was our limited number of biological samples (4 samples/tissue type) for the untargeted lipidomics analysis. Future studies will evaluate lipidomics changes with a greater number of tissue samples per group and will be analyzed using q-value statistical analysis to show variance across samples.

In conclusion, HFD feeding of different mouse models with varying degrees of PN and metabolic dysfunction produced significant remodeling of the sciatic nerve lipidome and aberrant mitochondrial bioenergetics. Of the three mouse strains (BL6, BTBR, BKS), HFD-fed BL6 mice develop large fiber and small fiber PN and metabolic dysfunction that most closely resembles the human condition. These animals showed significant changes in neutral lipids, phospholipids, lysophospholipids, and plasmalogen levels in the sciatic nerve. Both SM and LPE were significantly altered in sciatic nerves only in the HFD-fed BL6 animals, indicating the importance of these lipids for maintaining small fiber nerve function. Although plasma and liver lipids were significantly impacted by the HFD across all murine strains, the plasma and liver lipid changes were not biomarkers of PN. The loss of mitochondrial bioenergetics capacity in the sensory sural nerves from HFD-fed BL6 mice differed from HFD-fed BL6 DRG neurons, which showed increased ATP production, potentially as a compensatory mechanism to restore ATP production distally in the injured nerve. Future studies will focus on determining lipid changes that damage specific subsets of nerve fibers, including small and large nerve fibers, as a potential pathogenic mechanism underlying specific PN phenotypes. Additionally, identifying the specific lipid species that drive mitochondrial dysfunction and nerve damage may provide novel therapeutic targets for PN associated with prediabetes and T2D.

## Data Availability

The original contributions presented in the study are included in the article/Supplementary Material, further inquiries can be directed to the corresponding author. Raw lipidomics data files are publicly available at the DOI: 10.5281/zenodo.6814022.

## Abbreviations

CL, cardiolipin; DG, diacylglycerol; DGAT2, diacylglycerol acyltransferase 2; DRG, dorsal root ganglion; FCCP, carbonyl cyanide-4-(trifluoromethoxy)phenyl-hydrazone; HFD, high-fat diet; IENFD, intraepidermal nerve fiber density; LC-MS/MS, liquid chromatography-tandem mass spectrometry; LPC, lysophosphatidylcholine; LPE, lysophosphatidylethanolamine; NCV, nerve conduction velocity; PC, phosphatidylcholine; PE, phosphatidylethanolamine; PI, phosphatidylinositol; plasmenyl-PE, plasmenyl-phosphatidylethanolamine; plasmenyl-PC, plasmenylphosphatidylcholine; PLS-DA, Partial least squares-discriminant analysis; PN, peripheral neuropathy; PS, phosphatidylserine; SM, sphingomyelin; TG, triglyceride; T2D, type 2 diabetes; VIP, variable importance in projection.

## References

[B1] Ackerman D. Tumanov S. Qiu B. Michalopoulou E. Spata M. Azzam A. (2018). Triglycerides Promote Lipid Homeostasis during Hypoxic Stress by Balancing Fatty Acid Saturation. Cell Rep. 24 (10), 2596–2605. e2595. 10.1016/j.celrep.2018.08.015 30184495 PMC6137821

[B2] Afshinnia F. Nair V. Lin J. Rajendiran T. M. Soni T. Byun J. (2019). Increased Lipogenesis and Impaired β-oxidation Predict Type 2 Diabetic Kidney Disease Progression in American Indians. JCI Insight 4 (21). 10.1172/jci.insight.130317 PMC6948762 31573977

[B3] Afshinnia F. Rajendiran T. M. Soni T. Byun J. Wernisch S. Sas K. M. (2018). Impaired β-Oxidation and Altered Complex Lipid Fatty Acid Partitioning with Advancing CKD. J. Am. Soc. Nephrol. 29 (1), 295–306. 10.1681/ASN.2017030350 29021384 PMC5748913

[B4] Akoumi A. Haffar T. Mousterji M. Kiss R. S. Bousette N. (2017). Palmitate Mediated Diacylglycerol Accumulation Causes Endoplasmic Reticulum Stress, Plin2 Degradation, and Cell Death in H9C2 Cardiomyoblasts. Exp. Cell Res. 354 (2), 85–94. 10.1016/j.yexcr.2017.03.032 28336294

[B5] Ampong I. John Ikwuobe O. Brown J. E. P. Bailey C. J. Gao D. Gutierrez-Merino J. (2022). Odd Chain Fatty Acid Metabolism in Mice after a High Fat Diet. Int. J. Biochem. Cell Biol. 143, 106135. 10.1016/j.biocel.2021.106135 34896612 PMC8811477

[B6] Andersen S. T. Witte D. R. Dalsgaard E.-M. Andersen H. Nawroth P. Fleming T. (2018). Risk Factors for Incident Diabetic Polyneuropathy in a Cohort with Screen-Detected Type 2 Diabetes Followed for 13 Years: ADDITION-Denmark. Diabetes Care 41 (5), 1068–1075. 10.2337/dc17-2062 29487078

[B7] Aon M. A. Bhatt N. Cortassa S. C. (2014). Mitochondrial and Cellular Mechanisms for Managing Lipid Excess. Front. Physiol. 5, 282. 10.3389/fphys.2014.00282 25132820 PMC4116787

[B8] Bailey A. P. Koster G. Guillermier C. Hirst E. M. A. MacRae J. I. Lechene C. P. (2015). Antioxidant Role for Lipid Droplets in a Stem Cell Niche of Drosophila. Cell 163 (2), 340–353. 10.1016/j.cell.2015.09.020 26451484 PMC4601084

[B9] Basu Ball W. Neff J. K. Gohil V. M. (2018). The Role of Nonbilayer Phospholipids in Mitochondrial Structure and Function. FEBS Lett. 592 (8), 1273–1290. 10.1002/1873-3468.12887 29067684 PMC5918238

[B10] Brereton R. G. L. Lloyd G. R. (2014). Partial Least Squares Discriminant Analysis: Taking the Magic Away. J. Chemom. 28 (4), 213–225.

[B11] Calderon R. O. Attema B. DeVries G. H. (1995). Lipid Composition of Neuronal Cell Bodies and Neurites from Cultured Dorsal Root Ganglia. J. Neurochem. 64 (1), 424–429. 10.1046/j.1471-4159.1995.64010424.x 7798942

[B12] Callaghan B. C. Feldman E. Liu J. Kerber K. Pop-Busui R. Moffet H. (2011). Triglycerides and Amputation Risk in Patients with Diabetes. Diabetes Care 34 (3), 635–640. 10.2337/dc10-0878 21285390 PMC3041196

[B13] Callaghan B. C. Xia R. Banerjee M. de Rekeneire N. Harris T. B. Newman A. B. (2016a). Metabolic Syndrome Components Are Associated with Symptomatic Polyneuropathy Independent of Glycemic Status. Diabetes Care 39 (5), 801–807. 10.2337/dc16-0081 26965720 PMC4839175

[B14] Callaghan B. C. Xia R. Reynolds E. Banerjee M. Rothberg A. E. Burant C. F. (2016b). Association between Metabolic Syndrome Components and Polyneuropathy in an Obese Population. JAMA Neurol. 73 (12), 1468–1476. 10.1001/jamaneurol.2016.3745 27802497 PMC5217829

[B15] Cho H. W. Kim S. B. Jeong M. K. Park Y. Miller N. G. Ziegler T. R. (2008). Discovery of Metabolite Features for the Modelling and Analysis of High-Resolution NMR Spectra. Int. J. Data Min. Bioinform 2 (2), 176–192. 10.1504/ijdmb.2008.019097 18767354 PMC3883573

[B16] Chowdhury S. K. R. Smith D. R. Fernyhough P. (2013). The Role of Aberrant Mitochondrial Bioenergetics in Diabetic Neuropathy. Neurobiol. Dis. 51, 56–65. 10.1016/j.nbd.2012.03.016 22446165

[B17] Eichberg J. Zhu X. (1992). Diacylglycerol Composition and Metabolism in Peripheral Nerve. Adv. Exp. Med. Biol. 318, 413–425. 10.1007/978-1-4615-3426-6_37 1636507

[B18] Falabella M. Vernon H. J. Hanna M. G. Claypool S. M. Pitceathly R. D. S. (2021). Cardiolipin, Mitochondria, and Neurological Disease. Trends Endocrinol. Metabolism 32 (4), 224–237. 10.1016/j.tem.2021.01.006 PMC8277580 33640250

[B19] Feldman E. L. Callaghan B. C. Pop-Busui R. Zochodne D. W. Wright D. E. Bennett D. L. (2019). Diabetic Neuropathy. Nat. Rev. Dis. Prim. 5 (1), 41. 10.1038/s41572-019-0092-1 31197153

[B20] Feldman E. L. Nave K.-A. Jensen T. S. Bennett D. L. H. (2017). New Horizons in Diabetic Neuropathy: Mechanisms, Bioenergetics, and Pain. Neuron 93 (6), 1296–1313. 10.1016/j.neuron.2017.02.005 28334605 PMC5400015

[B21] Festing M. F. W. Altman D. G. (2002). Guidelines for the Design and Statistical Analysis of Experiments Using Laboratory Animals. ILAR J. 43 (4), 244–258. 10.1093/ilar.43.4.244 12391400

[B22] Galindo-Prieto B. Eriksson L. Trygg J. (2014). Variable Influence on Projection (VIP) for Orthogonal Projections to Latent Structures (OPLS). J. Chemom. 28 (8), 623–632. 10.1002/cem.2627

[B23] Ghosh S. Basu Ball W. Madaris T. R. Srikantan S. Madesh M. Mootha V. K. (2020). An Essential Role for Cardiolipin in the Stability and Function of the Mitochondrial Calcium Uniporter. Proc. Natl. Acad. Sci. U.S.A. 117 (28), 16383–16390. 10.1073/pnas.2000640117 32601238 PMC7368250

[B24] Guo K. Savelieff M. G. Rumora A. E. Alakwaa F. M. Callaghan B. C. Hur J. (2021). Plasma Metabolomics and Lipidomics Differentiate Obese Individuals by Peripheral Neuropathy Status. J. Clin. Endocrinol. Metab. 107, 1091–1109. 10.1210/clinem/dgab844 PMC8947234 34878536

[B25] Hinder L. M. O'Brien P. D. Hayes J. M. Backus C. Solway A. P. Sims-Robinson C. (2017). Dietary Reversal of Neuropathy in a Murine Model of Prediabetes and the Metabolic Syndrome. Dis. Model Mech. 10 (6), 717–725. 10.1242/dmm.028530 28381495 PMC5483005

[B26] Hinder L. M. Vincent A. M. Burant C. F. Pennathur S. Feldman E. L. (2012). Bioenergetics in Diabetic Neuropathy: what We Need to Know. J. Peripher Nerv. Syst. 17 (Suppl. 2), 10–14. 10.1111/j.1529-8027.2012.00389.x 22548617 PMC3589977

[B27] Hornemann T. (2021). Mini Review: Lipids in Peripheral Nerve Disorders. Neurosci. Lett. 740, 135455. 10.1016/j.neulet.2020.135455 33166639

[B28] Inoue M. Xie W. Matsushita Y. Chun J. Aoki J. Ueda H. (2008). Lysophosphatidylcholine Induces Neuropathic Pain through an Action of Autotaxin to Generate Lysophosphatidic Acid. Neuroscience 152 (2), 296–298. 10.1016/j.neuroscience.2007.12.041 18280050

[B29] Kristensen F. P. Christensen D. H. Callaghan B. C. Kahlert J. Knudsen S. T. Sindrup S. H. (2020). Statin Therapy and Risk of Polyneuropathy in Type 2 Diabetes: A Danish Cohort Study. Diabetes Care 43 (12), 2945–2952. 10.2337/dc20-1004 32998990

[B30] Kuge H. Akahori K. Yagyu K.-i. Honke K. (2014). Functional Compartmentalization of the Plasma Membrane of Neurons by a Unique Acyl Chain Composition of Phospholipids. J. Biol. Chem. 289 (39), 26783–26793. 10.1074/jbc.M114.571075 25096572 PMC4175321

[B31] Liu Y. Thalamuthu A. Mather K. A. Crawford J. Ulanova M. Wong M. W. K. (2021). Plasma Lipidome Is Dysregulated in Alzheimer's Disease and Is Associated with Disease Risk Genes. Transl. Psychiatry 11 (1), 344. 10.1038/s41398-021-01362-2 34092785 PMC8180517

[B32] Llano D. A. Devanarayan V. Alzheimer's Disease Neuroimaging I. (2021). Serum Phosphatidylethanolamine and Lysophosphatidylethanolamine Levels Differentiate Alzheimer's Disease from Controls and Predict Progression from Mild Cognitive Impairment. J. Alzheimers Dis. 80 (1), 311–319. 10.3233/JAD-201420 33523012

[B33] Lupachyk S. Watcho P. Hasanova N. Julius U. Obrosova I. G. (2012). Triglyceride, Nonesterified Fatty Acids, and Prediabetic Neuropathy: Role for Oxidative-Nitrosative Stress. Free Radic. Biol. Med. 52 (8), 1255–1263. 10.1016/j.freeradbiomed.2012.01.029 22366714 PMC3312982

[B34] Montgomery M. K. Hallahan N. L. Brown S. H. Liu M. Mitchell T. W. Cooney G. J. (2013). Mouse Strain-dependent Variation in Obesity and Glucose Homeostasis in Response to High-Fat Feeding. Diabetologia 56 (5), 1129–1139. 10.1007/s00125-013-2846-8 23423668

[B35] Murphy E. J. (2017). Ether Lipids and Their Elusive Function in the Nervous System: a Role for Plasmalogens. J. Neurochem. 143 (5), 463–466. 10.1111/jnc.14156 28944460

[B36] Murray A. J. Knight N. S. Little S. E. Cochlin L. E. Clements M. Clarke K. (2011). Dietary Long-Chain, but Not Medium-Chain, Triglycerides Impair Exercise Performance and Uncouple Cardiac Mitochondria in Rats. Nutr. Metab. (Lond) 8, 55. 10.1186/1743-7075-8-55 21806803 PMC3168416

[B37] National Diabetes Fact Sheet (2011). National Diabetes Fact Sheet. [Online]. Available at: http://www.cdc.gov/diabetes/pubs/pdf/ndfs_2011.pdf (accessed May 1, 2014).

[B38] Nishizuka Y. (1992). Intracellular Signaling by Hydrolysis of Phospholipids and Activation of Protein Kinase C. Science 258 (5082), 607–614. 10.1126/science.1411571 1411571

[B39] O'Brien P. D. Guo K. Eid S. A. Rumora A. E. Hinder L. M. Hayes J. M. (2020). Integrated Lipidomic and Transcriptomic Analyses Identify Altered Nerve Triglycerides in Mouse Models of Prediabetes and Type 2 Diabetes. Dis. Model Mech. 13 (2). 10.1242/dmm.042101 PMC6994925 31822493

[B40] O'Brien P. D. Hinder L. M. Callaghan B. C. Feldman E. L. (2017). Neurological Consequences of Obesity. Lancet Neurology 16 (6), 465–477. 10.1016/S1474-4422(17)30084-4 28504110 PMC5657398

[B41] Palavicini J. P. Chen J. Wang C. Wang J. Qin C. Baeuerle E. (2020). Early Disruption of Nerve Mitochondrial and Myelin Lipid Homeostasis in Obesity-Induced Diabetes. JCI Insight 5 (21). 10.1172/jci.insight.137286 PMC7710310 33148881

[B42] Paradies G. Paradies V. De Benedictis V. Ruggiero F. M. Petrosillo G. (2014). Functional Role of Cardiolipin in Mitochondrial Bioenergetics. Biochimica Biophysica Acta (BBA) - Bioenergetics 1837 (4), 408–417. 10.1016/j.bbabio.2013.10.006 24183692

[B43] Poitelon Y. Kopec A. M. Belin S. (2020). Myelin Fat Facts: An Overview of Lipids and Fatty Acid Metabolism. Cells 9 (4), 812. 10.3390/cells9040812 PMC7226731 32230947

[B44] Pooya S. Liu X. Kumar V. B. S. Anderson J. Imai F. Zhang W. (2014). The Tumour Suppressor LKB1 Regulates Myelination through Mitochondrial Metabolism. Nat. Commun. 5, 4993. 10.1038/ncomms5993 25256100 PMC4431623

[B45] Rimola V. Hahnefeld L. Zhao J. Jiang C. Angioni C. Schreiber Y. (2020). Lysophospholipids Contribute to Oxaliplatin-Induced Acute Peripheral Pain. J. Neurosci. 40 (49), 9519–9532. 10.1523/JNEUROSCI.1223-20.2020 33158961 PMC7724144

[B46] Rohart F. Gautier B. Singh A. Lê Cao K.-A. (2017). mixOmics: An R Package for 'omics Feature Selection and Multiple Data Integration. PLoS Comput. Biol. 13 (11), e1005752. 10.1371/journal.pcbi.1005752 29099853 PMC5687754

[B47] Rumora A. E. Guo K. Alakwaa F. M. Andersen S. T. Reynolds E. L. Jørgensen M. E. (2021). Plasma Lipid Metabolites Associate with Diabetic Polyneuropathy in a Cohort with Type 2 Diabetes. Ann. Clin. Transl. Neurol. 8 (6), 1292–1307. 10.1002/acn3.51367 33955722 PMC8164865

[B48] Rumora A. E. Lentz S. I. Hinder L. M. Jackson S. W. Valesano A. Levinson G. E. (2018). Dyslipidemia Impairs Mitochondrial Trafficking and Function in Sensory Neurons. FASEB J. 32 (1), 195–207. 10.1096/fj.201700206R 28904018 PMC6191072

[B49] Rumora A. E. LoGrasso G. Haidar J. A. Dolkowski J. J. Lentz S. I. Feldman E. L. (2019a). Chain Length of Saturated Fatty Acids Regulates Mitochondrial Trafficking and Function in Sensory Neurons. J. Lipid Res. 60 (1), 58–70. 10.1194/jlr.M086843 30442656 PMC6314260

[B50] Rumora A. E. LoGrasso G. Hayes J. M. Mendelson F. E. Tabbey M. A. Haidar J. A. (2019b). The Divergent Roles of Dietary Saturated and Monounsaturated Fatty Acids on Nerve Function in Murine Models of Obesity. J. Neurosci. 39 (19), 3770–3781. 10.1523/JNEUROSCI.3173-18.2019 30886017 PMC6510336

[B51] Sajic M. Rumora A. E. Kanhai A. A. Dentoni G. Varatharajah S. Casey C. (2021). High Dietary Fat Consumption Impairs Axonal Mitochondrial Function *In Vivo* . J. Neurosci. 41 (19), 4321–4334. 10.1523/jneurosci.1852-20.2021 33785643 PMC8143198

[B52] Sas K. M. Lin J. Rajendiran T. M. Soni T. Nair V. Hinder L. M. (2018). Shared and Distinct Lipid-Lipid Interactions in Plasma and Affected Tissues in a Diabetic Mouse Model. J. Lipid Res. 59 (2), 173–183. 10.1194/jlr.M077222 29237716 PMC5794414

[B53] Schenkel L. C. Bakovic M. (2014). Formation and Regulation of Mitochondrial Membranes. Int. J. Cell Biol. 2014–13. 10.1155/2014/709828 PMC3918842 24578708

[B54] Smith A. G. Singleton J. R. (2013). Obesity and Hyperlipidemia Are Risk Factors for Early Diabetic Neuropathy. J. Diabetes its Complicat. 27 (5), 436–442. 10.1016/j.jdiacomp.2013.04.003 PMC3766404 23731827

[B55] Surma M. A. Gerl M. J. Herzog R. Helppi J. Simons K. Klose C. (2021). Mouse Lipidomics Reveals Inherent Flexibility of a Mammalian Lipidome. Sci. Rep. 11 (1), 19364. 10.1038/s41598-021-98702-5 34588529 PMC8481471

[B56] Tan S. T. Ramesh T. Toh X. R. Nguyen L. N. (2020). Emerging Roles of Lysophospholipids in Health and Disease. Prog. Lipid Res. 80, 101068. 10.1016/j.plipres.2020.101068 33068601

[B57] Tasseva G. Bai H. D. Davidescu M. Haromy A. Michelakis E. Vance J. E. (2013). Phosphatidylethanolamine Deficiency in Mammalian Mitochondria Impairs Oxidative Phosphorylation and Alters Mitochondrial Morphology. J. Biol. Chem. 288 (6), 4158–4173. 10.1074/jbc.M112.434183 23250747 PMC3567666

[B58] Tracey T. J. Steyn F. J. Wolvetang E. J. Ngo S. T. (2018). Neuronal Lipid Metabolism: Multiple Pathways Driving Functional Outcomes in Health and Disease. Front. Mol. Neurosci. 11, 10. 10.3389/fnmol.2018.00010 29410613 PMC5787076

[B59] Troyanskaya O. Cantor M. Sherlock G. Brown P. Hastie T. Tibshirani R. (2001). Missing Value Estimation Methods for DNA Microarrays. Bioinformatics 17 (6), 520–525. 10.1093/bioinformatics/17.6.520 11395428

[B60] Venn-Watson S. Lumpkin R. Dennis E. A. (2020). Efficacy of Dietary Odd-Chain Saturated Fatty Acid Pentadecanoic Acid Parallels Broad Associated Health Benefits in Humans: Could it Be Essential? Sci. Rep. 10 (1), 8161. 10.1038/s41598-020-64960-y 32424181 PMC7235264

[B61] Wang H.-Y. Tsai Y.-J. Chen S.-H. Lin C.-T. Lue J.-H. (2013). Lysophosphatidylcholine Causes Neuropathic Pain via the Increase of Neuronal Nitric Oxide Synthase in the Dorsal Root Ganglion and Cuneate Nucleus. Pharmacol. Biochem. Behav. 106, 47–56. 10.1016/j.pbb.2013.03.002 23541495

[B62] Wang M. Xie M. Yu S. Shang P. Zhang C. Han X. (2021). Lipin1 Alleviates Autophagy Disorder in Sciatic Nerve and Improves Diabetic Peripheral Neuropathy. Mol. Neurobiol. 58, 6049–6061. 10.1007/s12035-021-02540-5 34435332

[B63] Wiggin T. D. Sullivan K. A. Pop-Busui R. Amato A. Sima A. A. F. Feldman E. L. (2009). Elevated Triglycerides Correlate with Progression of Diabetic Neuropathy. Diabetes 58 (7), 1634–1640. 10.2337/db08-1771 19411614 PMC2699859

[B64] Yang C. Wang X. Wang J. Wang X. Chen W. Lu N. (2020). Rewiring Neuronal Glycerolipid Metabolism Determines the Extent of Axon Regeneration. Neuron 105 (2), 276–292. 10.1016/j.neuron.2019.10.009 31786011 PMC6975164

[B65] Zhang M. Mileykovskaya E. Dowhan W. (2005). Cardiolipin Is Essential for Organization of Complexes III and IV into a Supercomplex in Intact Yeast Mitochondria. J. Biol. Chem. 280 (33), 29403–29408. 10.1074/jbc.M504955200 15972817 PMC4113954

