# Supplementary material for: A High-Fat Diet Disrupts Nerve Lipids and Mitochondrial Function in Murine Models of Neuropathy
Source: Front Physiol. 2022 Aug 22;13:921942. doi: 10.3389/fphys.2022.921942 (PMC9441493; doi:10.3389/fphys.2022.921942)
Supplement: Supplementary file 1 [file DataSheet1.docx]

Supplementary Material

**Supplemental Table 1.** Fatty acid composition of the standard diet (D12450B, 10% kcal fat, Research Diets) and 54% high fat diet (D05090701, 54% kcal fat from lard, Research Diets).

**Supplemental Table 2.** Internal standards for lipidomics analysis.

| Name | Abbreviation | CAS | FORMULA | Exact mass |
| --- | --- | --- | --- | --- |
|  |  |  |  |  |
| 1-heptadecanoyl-2-hydroxy-sn-glycero-3-phosphocholine | lysoPC 17:0 | 50930-23-9 | C25H52NO7P | 509.34814 |
| 1,2-diheptadecanoyl-sn-glycero-3-phosphocholine | PC 34:0 | 70897-27-7 | C42H84NO8P | 761.59345 |
| 1,2-diheptadecanoyl-sn-glycero-3-phosphoethanolamine | PE 34:0 | 140219-78-9 | C39H78NO8P | 719.54651 |
| 1,2-diheptadecanoyl-sn-glycero-3-phospho-L-serine | PS 34:0 | 799268-51-2 | C40H78NO10P | 763.53633 |
| N-heptadecanoyl-D-erythro-sphingosylphosphorylcholine | SM 35:1 | 121999-64-2 | C40H81N2O6P | 716.58322 |
| cholest-5-en-3ß-yl heptadecanoate | CE 17:0 | 24365-37-5 | C44H78O2 | 638.60018 |
| 1-palmitoyl-2-oleoyl-sn-glycerol | DG 34:1 | 29541-66-0 | C37H70O5 | 594.52232 |
| 1-heptadecanoyl-rac-glycerol | MG 17:0 | 5638-14-2 | C20H40O4 | 344.29266 |
| 1,2,3-triheptadecanoyl-glycerol | TG 51:0 |  | C54H104O6 | 848.78329 |
| N-heptadecanoyl-D-erythro-sphingosine | Ceramide | 67492-16-4 | C35H69NO3 | 551.52774 |
| 1,2-diheptadecanoyl-sn-glycero-3-phosphate | PA 34:0 | 154804-54-3 | C37H73O8P | 676.50431 |
| 1,2-diheptadecanoyl-sn-glycero-3-phospho-(1'-rac-glycerol) | PG 34:0 | 799268-52-3 | C40H79O10P | 750.541085 |
| 1,3(d5)-dinonadecanoyl-glycerol | 1,3-19:0 D5 DG | 1246523-66-9 | C41H75D5O5 | 657.632 |
| Glyceryl tri(hexadecanoate-d31) | D-31 TAG | 241157-04-0 | C51D93H5O6 | 900.89 |

**Supplemental Table 3.** Internal standard reproducibility negative.

| **Name** | **RSD%** |
| --- | --- |
| IS PC 34:0;[M-Ac-H]- | 22.26437 |
| IS PG 34:0 ;[M-H]- | 15.0556 |
| IS PI 37:4 ;[M-H]- | 23.12844 |
| IS PS 34:0 ;[M-H]- | 24.2379 |

**Supplemental Table 4.** Internal standard reproducibility positive.

| **Name** | **RSD%** |
| --- | --- |
| IS CE 17:0; [M+NH4]+ | 9.304357 |
| IS Ceramide;[M-H20]+ | 7.569589 |
| IS D31-TAG | 21.79915 |
| IS d5-DAG | 22.09684 |
| IS DG 34:1; [M+NH4]+ | 12.22205 |
| IS lysoPC 17:0;[M+H]+ | 2.603369 |
| IS MG 17:0; [M+NH4]+ | 8.411796 |
| IS PC 34:0; [M+H]+ | 12.12368 |
| IS PS 34:0;[M+H]+ | 22.10686 |
| IS SM 35:1;[M+H]+ | 7.417303 |

**Supplemental Table 5.** Neuropathy and metabolic measurements for 36-week-old BL6, BTBR, and BKS mice after SD vs. HFD feeding.

| Phenotype | BL6 | | BTBR | | BKS | |
| --- | --- | --- | --- | --- | --- | --- |
|  | SD | HFD | SD | HFD | SD | HFD |
| Neuropathy measurements | | | | | | |
| Sciatic NCVs (n=6) | 61 ± 0.96 | 46.67 ± 1.05*** | 60.2 ± 0.92 | 50.17 ± 0.70*** | 60.71 ± 0.78 | 54.83 ± 1.85** |
| Sural NCVs | 23.33 ± 0.21 | 20.17 ± 1.11** | 22 ± 0.37 | 17.5 ± 0.34*** | 22.71 ± 0.47 | 19.67 ± 0.56*** |
| IENFDs (n=7-9) | 39.04 ± 2.47 | 28.44 ± 2.02** | 37.16 ± 2.01 | 30.11 ± 1.51 | 22.71 ± 0.47 | 19.67 ± 0.56 |
| Longitudinal body weight | | | | | | |
| BW (grams)  8 weeks | 24.78 ± 0.31 | 25.77 ± 0.94 | 32.51 ± 0.53 | 35.38 ± 0.94 | 23.47 ± 0.3 | 22.54 ± 0.58 |
| BW (grams)  16 weeks | 28.8 ± 0.58 | 41.1 ± 1.67*** | 41.1 ± 0.8 | 44.45 ± 1.4 | 27.83 ± 0.53 | 29.38 ± 1.73 |
| BW (grams)  24 weeks | 33.58 ± 0.7 | 35.24 ± 2.43*** | 46.79 ± 0.94 | 48.99 ± 1.14 | 30.64 ± 0.89 | 31.06 ± 3.79 |
| BW (grams)  36 weeks | 36.07 ± 0.95 | 49.95 ± 1.74*** | 48.01 ± 0.93 | 57.12 ± 1.75*** | 29.85 ± 0.78 | 31.53 ± 2.4 |
| Additional metabolic measurements | | | | | | |
| Fasting glucose (mg/dl) | 229 ± 11 | 232 ± 11 | 186 ± 8 | 229 ± 11** | 211 ± 13 | 177 ± 15 |
| Plasma insulin (ng/ml) | 0.89 ± 0.3 | 5.36 ± 1.8* | 2.01 ± 0.4 | 12.25 ± 4.7* | 0.46 ± 0.1 | 1.58 ± 0.4* |
| Triglycerides (mg/dl) | 33.8 ± 4.5 | 23.3 ± 1.7 | 28.3 ± 1.1 | 28.3 ± 2.7 | 47.5 ± 9.6 | 33 ± 2.7 |
| Cholesterol (mg/dl) | 85.2 ± 16.5 | 176.7 ± 36.8* | 89.7 ± 10.5 | 84.5 ± 12.7 | 57 ± 9.6 | 74.5 ± 13.5 |
| Glycated hemoglobin (mmol/mol) [%GHb]  (n=10) | 6.5 ± 0.2 (48) | 6.5 ± 0.2 (48) | 7.6 ± 0.4 (60) | 7.3 ± 0.3 (56) | 5.8 ± 0.2 (40) | 6.4 ± 0.2 (46) |

Neuropathy measurements included sciatic nerve conduction velocity (NCV) (n=6), sural NCV (n=6), and intraepidermal nerve fiber density (IENFD) (n=7-9). Metabolic measurements included body weights (n=6-12), fasting glucose (n=6), plasma insulin (n=6), triglycerides (n=6), cholesterol (n=6), and glycated hemoglobin (n=10). *P<0.05, **P<0.01, ***P<0.001, HFD vs respective SD-fed controls. All neuropathy and metabolic measurements were previously collected by Hinder et al. (Hinder et al., 2017).

**Supplemental Figure 1.** Plasma **(A)** triglyceride (TG) **(B)** diacylglycerol (DG) and **(C)** cholesterol ester (CE) lipid changes for HFD BL6 and BTBR mice compared to SD BL6 and BTBR mice. There were no significantly different plasma TGs or DGs for BKS mice. *t*-test, P-value < 0.05.

**Supplemental Figure 2.** Plasma phospholipids for HFD BL6, BTBR, and BKS mice compared to SD BL6, BTBR, and BKS including **(A)** phosphatidylcholine (PC), **(B)** phosphatidylethanolamine (PE), **(C)** phosphatidylinositol (PI), **(D)** phosphatidylglycerol (PG), and **(E)** phosphatidic acid (PA). *t*-test, P-value < 0.05.

**Supplemental Figure 3.** Plasma cardiolipin (CL) **(A)** and sphingomyelin (SM) **(B)** changes in BL6, BTBR, and BKS mice fed the HFD versus SD. *t*-test, P-value < 0.05.

**Supplemental Figure 4.** Plasma plasmenylphospholipid and lysophospholipid levels in HFD BL6, BTBR, and BKS mice compared to SD BL6, BTBR, and BKS mice including **(A)** plasmenyl-phosphatidylethanolamine (plasmenyl-PE) **(B)** plasmenyl-phosphatidylcholine (plasmenyl-PC) **(C)** lysophosphatidylcholine (LPC) and **(D)** lysophosphatidylethanolamine (LPE). *t*-test, P-value < 0.05.

**Supplemental Figure 5.** Liver **(A)** triglycerides (TGs) **(B)** monoacylglycerols (MGs) **(C)** diacylglycerols (DGs) and **(D)** cholesterol ester (CE) lipids in HFD BL6 and BTBR mice compared to SD BL6 and BTBR mice. There were no significantly different plasma TGs or DGs for BKS mice. *t*-test, P-value < 0.05.

**Supplemental Figure 6.** Liver phospholipid levels in BL6, BTBR, and BKS mice fed HFD versus SD including **(A)** phosphatidylcholine (PC) **(B)** phosphatidylethanolamine (PE) **(C)** phosphatidylinositol (PI) **(D)** phosphatidylserine (PS) **(E)** phosphatidylglycerol (PG) and **(F)** phosphatidic acid (PA). *t*-test, P-value < 0.05.

**Supplemental Figure 7.** Liver cardiolipin (CL) and sphingomyelin (SM) levels in HFD BL6, BTBR, and BKS mice compared to SD BL6, BTBR, and BKS including **(A)** CL and **(B)** SM. *t*-test, P-value < 0.05.

**Supplemental Figure 8.** Liver plasmalogen and lysophospholipid levels in HFD BL6, BTBR, and BKS mice versus SD BL6, BTBR, and BKS including **(A)** plasmenyl-phosphatidylethanolamine (plasmenyl-PE), **(B)** plasmenyl-phosphatidylcholine (plasmenyl-PC), **(C)** lysophosphatidylcholine (LPC) and **(D)** lysophosphatidylethanolamine (LPE). *t*-test, P-value < 0.05.

| **Lipid** | **VIP scores** |
| --- | --- |
| PC 44:2 | 1.60574365 |
| plasmenyl-PE 36:1 | 1.607156955 |
| PS 36:2 | 1.611446815 |
| plasmenyl-PE 34:2 | 1.61708875 |
| PC 30:0 | 1.626489347 |
| TG 54:7 | 1.626759643 |
| TG 54:2 | 1.634735173 |
| PC 38:4 | 1.641212819 |
| CL 78:3 | 1.644303467 |
| PE 36:2 | 1.645624177 |
| TG 58:9 | 1.654591807 |
| plasmenyl-PE 40:2 | 1.658955908 |
| TG 54:1 | 1.660935679 |
| TG 56:8 | 1.661273581 |
| PC 44:1 | 1.663360146 |
| TG 56:9 | 1.664982845 |
| TG 54:5 | 1.676730786 |
| TG 52:1 | 1.699754985 |
| TG 54:6 | 1.705844629 |
| plasmenyl-PE 32:1 | 1.709382051 |

**Supplemental Table 6.** Variable importance in projection (VIP) scores of the top 20 PLS-DA lipids in sciatic nerve.

**Supplemental Table 7.** Variable importance in projection (VIP) scores of the top 20 PLS-DA lipids in liver.

| **Lipid** | **VIP scores** |
| --- | --- |
| PA 34:2 | 1.523375777 |
| TG 60:7 | 1.524805749 |
| PC 36:5 | 1.526766474 |
| DG 40:5 | 1.528013244 |
| PC 42:1 | 1.536507649 |
| CL 68:6 | 1.537412168 |
| PG 32:1 | 1.539216079 |
| lysoPE 16:1 | 1.546238206 |
| PE 32:1 | 1.546518436 |
| PE 38:3 | 1.548974277 |
| CL 78:9 | 1.552012813 |
| PE 34:1 | 1.554169186 |
| PC 32:1 | 1.555009755 |
| PE 32:2 | 1.559056489 |
| TG 60:11 | 1.565118788 |
| PE 42:7 | 1.565702369 |
| TG 64:14 | 1.573514991 |
| PS 38:5 | 1.575163017 |
| PE 36:0 | 1.578396624 |
| TG 60:10 | 1.588243086 |

**Supplemental Table 8.** Variable importance in projection (VIP) scores of the top 20 PLS-DA lipids in plasma.

| **Lipid** | **VIP scores** |
| --- | --- |
| lysoPC 18:3 | 1.640689058 |
| lysoPE 18:1 | 1.643082743 |
| SM 36:0 | 1.647682335 |
| lysoPC 18:1 | 1.648778373 |
| plasmenyl-PC 18:0 | 1.653975818 |
| PC 34:4 | 1.660023264 |
| SM 34:1 | 1.662414939 |
| lysoPE 16:1 | 1.679451005 |
| PC 38:7 | 1.683360451 |
| SM 38:1 | 1.695002869 |
| CE 18:2 | 1.695475303 |
| SM 40:1 | 1.702195203 |
| lysoPC 20:1 | 1.705310877 |
| plasmenyl-PC 20:0 | 1.714698343 |
| PC 40:10 | 1.715325971 |
| PC 40:6 | 1.716435207 |
| PE 38:4 | 1.727757872 |
| lysoPC 14:0 | 1.752298374 |
| SM 38:0 | 1.756851619 |
| lysoPC 16:1 | 1.758239191 |

**Supplemental Figure 9. (A)** Venn diagram of lipids shared between plasma, liver, and sciatic nerve (SCN) tissues. **(B)** Heat map of lipid species in plasma, liver, and SCN from SD-fed (indicated by the horizontal green bar) and HFD-fed (indicated by the horizontal blue bar) BL6 mice.

**Supplemental Figure 10.** Venn diagram showing shared HFD-induced lipid changes between the three strains of mice (BL6, BTBR, BKS). There were 33 lipid changes shared between all three murine strains. BL6 and BTBR mice shared 57 lipid changes, BL6 and BKS shared 20 lipid changes, and BTBR and BKS mice shared 32 lipid changes. The lipids represented in this Venn diagram are given in Supplemental Table 9.

**Supplemental Table 9.** Shared VIP lipids across murine strains.

|  | **Shared** | **BL6** | **BTBR** | **BKS** | **BL6 vs. BTBR** | **BL6 vs. BKS** | **BTBR vs. BKS** |
| --- | --- | --- | --- | --- | --- | --- | --- |
| **1** | **CL 66:3** | **CL 68:2** | **CL 70:5** | **CL 68:1** | **CL 74:1** | **DG 36:2** | **CL 74:3** |
| **2** | **CL 74:4** | **CL 68:3** | **CL 78:0** | **CL 74:7** | **CL 76:1** | **DG 42:1** | **CL 74:5** |
| **3** | **CL 74:9** | **CerP 44:2** | **DG 36:4** | **CL 78:12** | **CL 78:3** | **DG 42:5** | **CL 80:1** |
| **4** | **DG 30:1** | **DG 32:0** | **DG 38:5** | **CL 80:9** | **CL 78:9** | **MG 18:1** | **DG 30:2** |
| **5** | **DG 40:0** | **DG 34:0** | **PC 34:0** | **CL 82:11** | **DG 36:1** | **PC 34:1** | **DG 32:1** |
| **6** | **DG 42:0** | **DG 36:0** | **PC 34:2** | **CL 82:9** | **DG 38:3** | **PC 34:3** | **DG 32:2** |
| **7** | **PC 32:1** | **DG 36:3** | **PC 34:4** | **DG 34:2** | **DG 38:4** | **PC 42:5** | **DG 34:3** |
| **8** | **PC 38:3** | **DG 38:0** | **PC 36:1** | **DG 38:7** | **DG 40:4** | **PC 44:1** | **PC 32:4** |
| **9** | **PC 40:2** | **DG 38:1** | **PC 36:4** | **DG 42:3** | **DG 42:2** | **PE 44:1** | **PC 38:6** |
| **10** | **PC 40:4** | **DG 40:1** | **PC 38:1** | **MG 16:0** | **DG 42:4** | **PG 36:1** | **PC 38:7** |
| **11** | **PE 32:2** | **DG 40:2** | **PC 38:5** | **MG 18:2** | **PC 28:0** | **PS 36:2** | **PC 40:8** |
| **12** | **PE 36:5** | **DG 40:5** | **PC 40:0** | **PA 34:2** | **PC 30:0** | **PS 38:6** | **PE 36:3** |
| **13** | **PS 36:3** | **DG 44:2** | **PC 40:1** | **PA 38:2** | **PC 30:1** | **SM 36:2** | **PE 36:4** |
| **14** | **PS 42:2** | **MG 18:0** | **PC 40:5** | **PA 42:4** | **PC 32:2** | **TG 52:5** | **PE 38:1** |
| **15** | **PS 44:3** | **PA 40:6** | **PC 42:0** | **PA 44:1** | **PC 36:5** | **TG 56:9** | **PE 40:2** |
| **16** | **SM 40:2** | **PC 32:0** | **PE 34:0** | **PC 36:2** | **PC 44:2** | **TG 58:3** | **PG 32:0** |
| **17** | **TG 42:0** | **PC 32:3** | **PE 36:0** | **PC 36:6** | **PE 32:1** | **TG 58:4** | **PG 36:0** |
| **18** | **TG 46:1** | **PC 36:3** | **PE 36:1** | **PC 40:3** | **PE 34:2** | **TG 58:6** | **PG 40:8** |
| **19** | **TG 48:2** | **PC 38:0** | **PE 38:3** | **PC 42:3** | **PE 34:3** | **TG 60:4** | **PI 36:1** |
| **20** | **TG 48:3** | **PC 38:4** | **PE 40:5** | **PC 42:4** | **PE 36:2** | **plasmenyl-PE 36:5** | **PI 38:3** |
| **21** | **TG 50:3** | **PC 38:8** | **PE 40:7** | **PC 42:9** | **PE 38:2** |  | **PS 42:5** |
| **22** | **TG 50:4** | **PC 42:10** | **PG 38:3** | **PC 44:4** | **PE 38:5** |  | **SM 36:1** |
| **23** | **TG 52:1** | **PC 42:2** | **PI 34:1** | **PC 48:4** | **PE 42:2** |  | **TG 42:1** |
| **24** | **TG 54:1** | **PC 44:12** | **PI 34:2** | **PE 32:0** | **PI 36:2** |  | **TG 42:2** |
| **25** | **TG 54:2** | **PC 46:6** | **PI 36:3** | **PE 42:1** | **PI 38:4** |  | **TG 44:1** |
| **26** | **TG 54:6** | **PE 40:3** | **PI 38:6** | **PE 42:5** | **PI 40:6** |  | **TG 44:2** |
| **27** | **TG 54:7** | **PE 40:4** | **PS 36:1** | **PE 44:10** | **PS 38:2** |  | **TG 46:2** |
| **28** | **TG 56:6** | **PE 42:3** | **PS 38:3** | **PE 44:3** | **PS 42:1** |  | **TG 46:3** |
| **29** | **TG 56:7** | **PE 44:2** | **PS 38:4** | **PG 34:1** | **TG 54:5** |  | **TG 50:5** |
| **30** | **TG 56:8** | **PE 46:1** | **PS 40:1** | **PG 36:4** | **TG 56:5** |  | **TG 54:4** |
| **31** | **TG 58:9** | **PE 46:4** | **PS 40:6** | **PG 38:4** | **TG 58:5** |  | **TG 56:3** |
| **32** | **lysoPC 16:1** | **PS 36:4** | **PS 40:7** | **PG 38:6** | **lysoPC 14:0** |  | **TG 58:8** |
| **33** | **lysoPC 26:0** | **PS 40:2** | **PS 42:4** | **PI 36:4** | **lysoPC 16:0** |  |  |
| **34** |  | **PS 44:12** | **SM 36:0** | **PI 38:5** | **lysoPC 18:1** |  |  |
| **35** |  | **SM 32:1** | **SM 38:1** | **PI 40:5** | **lysoPC 20:1** |  |  |
| **36** |  | **SM 34:2** | **lysoPC 20:4** | **PS 34:2** | **lysoPC 20:3** |  |  |
| **37** |  | **SM 42:2** | **lysoPC 22:5** | **PS 40:5** | **lysoPC 22:1** |  |  |
| **38** |  | **TG 52:6** | **lysoPE 18:2** | **PS 44:2** | **lysoPC 22:6** |  |  |
| **39** |  | **TG 54:3** | **lysoPE 24:0** | **SM 34:0** | **lysoPC 24:1** |  |  |
| **40** |  | **TG 56:2** | **plasmenyl-PE 30:0** | **SM 40:1** | **lysoPC 26:1** |  |  |
| **41** |  | **TG 56:4** | **plasmenyl-PE 36:0** | **SM 40:4** | **lysoPE 16:0** |  |  |
| **42** |  | **TG 62:4** | **plasmenyl-PE 36:4** | **SM 42:4** | **lysoPE 16:1** |  |  |
| **43** |  | **lysoPC 20:0** | **plasmenyl-PE 38:0** | **TG 40:0** | **lysoPE 18:1** |  |  |
| **44** |  | **lysoPC 22:0** | **plasmenyl-PE 40:6** | **TG 46:0** | **lysoPE 22:1** |  |  |
| **45** |  | **lysoPC 24:0** | **plasmenyl-PE 42:6** | **TG 48:0** | **lysoPE 22:6** |  |  |
| **46** |  | **lysoPE 14:0** |  | **TG 48:1** | **lysoPE 24:4** |  |  |
| **47** |  | **lysoPE 20:1** |  | **TG 50:1** | **plasmenyl-PC 18:0** |  |  |
| **48** |  | **lysoPE 20:3** |  | **TG 50:2** | **plasmenyl-PE 32:1** |  |  |
| **49** |  | **lysoPE 20:4** |  | **TG 52:2** | **plasmenyl-PE 34:1** |  |  |
| **50** |  | **lysoPE 22:4** |  | **TG 52:3** | **plasmenyl-PE 34:2** |  |  |
| **51** |  | **lysoPE 24:1** |  | **TG 52:4** | **plasmenyl-PE 34:3** |  |  |
| **52** |  | **plasmenyl-PE 18:0** |  | **lysoPE 18:3** | **plasmenyl-PE 36:1** |  |  |
| **53** |  | **plasmenyl-PE 20:0** |  | **plasmenyl-PE 36:3** | **plasmenyl-PE 36:2** |  |  |
| **54** |  | **plasmenyl-PE 22:0** |  | **plasmenyl-PE 38:4** | **plasmenyl-PE 38:1** |  |  |
| **55** |  | **plasmenyl-PE 40:1** |  | **plasmenyl-PE 40:5** | **plasmenyl-PE 38:2** |  |  |
| **56** |  | **plasmenyl-PE 40:4** |  | **plasmenyl-PE 42:4** | **plasmenyl-PE 38:5** |  |  |
| **57** |  |  |  |  | **plasmenyl-PE 40:2** |  |  |

**References:**

Hinder, L.M., O'Brien, P.D., Hayes, J.M., Backus, C., Solway, A.P., Sims-Robinson, C., et al. (2017). Dietary reversal of neuropathy in a murine model of prediabetes and metabolic syndrome. *Dis Model Mech* 10(6)**,** 717-725. doi: 10.1242/dmm.028530.
